# ﻿Nomenclature and typification in *Verbascum* (Scrophulariaceae) from North Africa

**DOI:** 10.3897/phytokeys.225.99356

**Published:** 2023-04-27

**Authors:** Hamid Khamar, Jalal El Oualidi, Amina Ouazzani Touhami, Laure Civeyrel

**Affiliations:** 1 Department of Botany and Plant Ecology, Scientific Institute, University Mohammed V in Rabat, B. P. 703, Rabat 10106, Morocco University Mohammed V in Rabat Rabat Morocco; 2 Plant, Animal Productions and Agro-industry Laboratory, Department of Biology, Faculty of Sciences, Ibn Tofail University, B.P. 133 14000, Kenitra, Morocco Ibn Tofail University Kénitra Morocco; 3 Laboratoire Évolution & Diversité Biologique (EDB UMR 5174), Université de Toulouse, CNRS, IRD, UPS. 118 route de Narbonne, Bat 4R1, 31062 Toulouse cedex 9, France Université de Toulouse Toulouse France

**Keywords:** genus *Verbascum*, lectotypification, nomenclature, North Africa

## Abstract

The progress of taxonomic work on native *Verbascum* L. taxa found in Morocco led to a search for reference specimens in various herbaria. This process was extended to the taxa found in the other four countries of North Africa (Algeria, Tunisia, Libya, and Egypt), which make up the southern shore of the Mediterranean basin. Numerous names were identified as needing typification or requiring corrections of their earlier lectotypifications in order to stabilize their nomenclature and provide a better definition of each taxon. As a result, lectotypes are now designated for 35 names, a neotype is proposed for *V.ballii* (Batt.) Hub.-Mor., and second-step lectotypes are proposed for V.faureisubsp.acanthifolium (Pau) Benedí & J.M.Monts. and *V.pinnatisectum* (Batt.) Benedí. Comments have been added for each typified name. Known isolectotypes are also mentioned whenever possible. Furthermore, some new combinations are proposed in this paper, namely V.longirostrevar.antiatlantica (Emb.) Khamar, **comb. nov.**, V.longirostrevar.atlantica (Maire) Khamar, **comb. nov.**, and V.longirostrevar.hoggarica (Maire) Khamar, **comb. nov.**

## ﻿Introduction

*Verbascum* L. (including *Celsia* L.) is a diverse genus of the figwort family Scrophulariaceae*sensu stricto*, tribe Scrophularieae (*sensu* Angiosperm Phylogeny Group (APG-IV) 2016), including 497 taxa (465 species and 32 subspecies) (Hassler 2022; Khamar 2022) broadly distributed throughout the Old World, particularly in the temperate northern hemisphere (Murbeck 1925, 1933; Huber-Morath 1978; Remal 2014; Sotoodeh et al. 2014, 2015; Zografidis 2016; Khamar et al. 2017). It reaches its highest species diversity in the Mediterranean region (Yılmaz and Dane 2012; Remal 2014; Sotoodeh 2015; Zografidis 2016) where 70% of all species can be found (Murbeck 1939; Sharifnia 2007; Benedí 2009; Catara et al. 2016). Outside its natural distribution area, the genus is naturalized in other regions around the world, *i.e*. the United States of America, Canada, South Africa, Hawaii, Mexico, Chile, Hispaniola, Argentina, Australia, New Zealand, the Indian Ocean island of La Reunion, and Japan (Parham and Healy 1976; Gross and Werner 1978; Gross 1980; Reinartz 1984; Juvik and Juvik 1993; Mito and Uesugi 2004; Baret et al. 2006; Durán-Espinosa 2006; Williams 2010; Alba 2011; Nesom 2019; Jaca 2017; Scaramuzzino et al. 2018). Further progress on the taxonomy of *Verbascum* has recently been made thanks to botanical studies, especially molecular studies in the north of the Mediterranean basin and in the Irano-Turanian biogeographic region (Al-Hadeethy et al. 2014; Ghahremaninejad et al. 2014; Remal 2014; Sotoodeh 2015). The results of these studies support the monophyly of the genus and its synonymy with *Celsia* L. However, the morphological infrageneric classifications proposed by Murbeck (1925, 1933) and Huber-Morath (1978) are not consistent with the molecular phylogeny.

**Figure 1.**
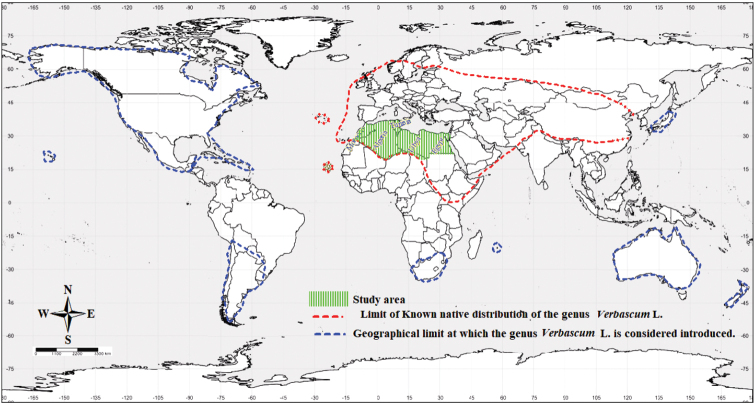
General distribution area of the Genus Verbascum L. modified from Murbeck (1925, 1933, 1939), Remal (2014), Sotoodeh et al. (2015) and Khamar (2022).

In continental North Africa (Fig. 1), the genus is represented by 36 taxa belonging to 31 species (Dobignard and Chatelain 2013; Khamar et al. 2017, 2022). Endemism rate is estimated to be 48% of all taxa. *Verbascum* species of North Africa grow in various habitats, i.e. steppes, forests, scrublands, lowland and high mountain pastures, rocky places, dry stony ravines and wadis, and can be found from the seashore up to high mountains (Jahandiez and Maire 1934; Quézel and Santa 1963; Pottier-Alapetite 1981; Qaiser 1982; Boulos 2002; Ibn Tattou 2007).

In continental North Africa, the genus has been studied by different authors from the late 18^th^ / early 19^th^ centuries to the present, and various accounts have been made: Desfontaines (1798); Ball (1875, 1878); Ascherson and Schweinfurth (1887); Battandier and Trabut (1889, 1890, 1902); Battandier (1910, 1919); Bonnet and Barratte (1896); Murbeck (1905, 1921, 1923, 1925, 1927, 1933, 1936, 1939); Maire (1918, 1924, 1931, 1940); Pau (1922, 1928); Sennen (1936); Jahandiez and Maire (1934); Emberger and Maire (1941); Quézel (1957); Quézel and Santa (1963); Huber-Morath (1973); Pottier-Alapetite, (1981); Qaiser (1982); Fennane and Ibn Tattou (1998); Boulos (2002); Ibn Tattou (2007); Le Floc’h et al. (2010); Dobignard and Chatelain (2013); and Khamar et al. (2017). Nevertheless, few papers specifically deal with the typification of accepted *Verbascum* species names (Benedí and Montserrat 1985, 1997; Benedí 2003), so that significant numbers of binomials still lack unequivocal types. Moreover, the locations of the types are not fully resolved.

The present paper constitutes a preliminary step towards a taxonomic and nomenclatural revision of the taxa growing in North Africa. The purpose is to (1) contribute to nomenclatural stability of the species by clarifying the type citations, the designation of lectotypes, holotypes or neotypes when necessary, or the indication of previous typifications, and (2) provide an updated list of synonyms for each taxon.

## ﻿Materials and methods

All the protologues of each validly published name for *Verbascum* taxa occurring in continental North Africa, as well as their synonyms, were consulted and critically reviewed. The biogeographic area was considered to extend from the southern shore of the Mediterranean Sea to the southern phytogeographic limit of the genus *Verbascum*, which is marked in southwestern Morocco by the presence of *V.sinuatum* L., *V.tetrandrum* Barr and Murb, *V.maroccanum* (Ball.) Huber-Morath and *V.longirostre* (Murb.) Huber-Morath; by *V.dentifolium* Delile and *V.longirostre* (Murb.) Huber-Morath in the Algerian Sahara (Hoggar in southern Algeria), by *V.tibesticum* (Quézel) Hub.-Mor. in the northern part of the Tibesti mountain range in Chad near the Chadian-Libyan border (Fig. 1).

Three key elements were taken into account to verify the original type plants (Turland et al. 2018): (1) the specimen characteristics that matched those in the original description, (2) the date and locality of collection mentioned in the protologue, and (3) all handwritten annotations on the labels.

Type herbarium specimens and authentic collections were examined, from the corresponding herbaria or mainly from the material loaded on the Global Biodiversity Information Facility (GBIF; available online at http://www.gbif.org/occurence), JSTOR Global Plant Science (available online at http://plants.jstor.org), and also from online access to the permanent websites of herbaria (in bold are indicated herbarium codes according to Thiers 2021, continuously updated, and listed in alphabetic order): **AIX** (available online via the P website link), **B** (available online at: http://ww2.bgbm.org/herbarium/default.cfm), **BC** (via the GBIF and JSTOR Global Plants websites), **BCN** (available online via GBIF and JSTOR Globl Plants website links), **BM** (available online via GBIF and JSTOR Global Plants website links), **COI** (available online at https://www.uc.pt/en/herbario_digital/catalogues), **G** (available online at http://www.ville-ge.ch/musinfo/bd/cjb/chg/index.php?lang=en), **GDA** (available online via the GBIF website link), **JE** (available online at https://herbarium.univie.ac.at/database/search.php), **K** (available online at http://apps.kew.org/herbcat/gotoSearchPage.do), **LD** (available online at https://www.biomus.lu.se/en/botanical-collections), **LINN** (available online at http://linnean-online.org/linnaean_herbarium.html), **MA** (available online via the GBIF website link), **MAF** (available online via the GBIF website link), **MPU** (available online at https://collections.umontpellier.fr/collections/botanique/herbier-mpu/base-herbier-mpu), **P** (available online at https://science.mnhn.fr/institution/mnhn/search), **RAB** (available online via P website and JSTOR Global Plants website links), and **S** (available online at http://herbarium.nrm.se/search/specimens/). The herbaria where the personal collection of each author is kept were often verified using the Taxonomic Literature of Stafleu and Cowan (1976–1988).

Under the guidance of the International Code of Nomenclature for algae, fungi, and plants (ICN; Turland et al. 2018) and statements recently suggested by McNeill (2014), the lectotype was selected among others according to its quality and its accordance with the description and data provided in the protologue. When duplicates were traced in other herbaria, these were designated as isolectotypes. When no appropriate material for use as a lectotype was located, a neotype was designated and its duplicates – if available – were designated as isoneotypes.

The following websites were also checked to collect more data about types, bibliographical citations in the original publications, names, and synonymies: the Euro + Med PlantBase (http://ww2.bgbm.org/EuroPlusMed/query.asp), the International Plant Name Index (IPNI 2022; http://www.ipni.org), Plants of the World Online (POWO 2022; http://www.plantsoftheworldonline.org/), Tropicos (http://www.tropicos.prg/Home.aspx), and the African Plant Database (2022; APD; https://africanplantdatabase.ch/).

## ﻿Lectotypifications

The taxa covered in this study are sorted in alphabetical order, according to their current accepted name (in bold), followed by the author citation, the bibliographic reference of the protologue or the nomenclatural recombination, the transcription of the original label of the specimen designated as type (lectotype, isolectotype, or neotype), and a barcode number following the herbarium acronym whenever available. Moreover, the homotypic and/or heterotypic synonyms for each name are quoted in chronological order. Comments have been added for each typified name.

### ﻿ Verbascum atlanticum

Taxon classificationPlantaeLamialesScrophulariaceae

Batt., in Battandier & Trabut, Fl. Algèrie, Dicot.: 626. 1889.

5BA93B95-243B-56E1-ADFC-B8B085FBCF25

= Verbascumrepandum Batt., in Suppl. aux Phanérog.: 69. 1910. Nonæ Willd. Enum. pl. hort. Berol.: 226, 1809.
= Verbascumpseudoblattaria auct., (sensu Batt) in Contrib. Fl. Atl.: 62. 1919., non. pseudoblattaria Schleicher, in Cat. Pl. Helv. ed. 4: 36. 1821. Type: [Algeria]. O. Djebel-M’zi, [without date], *J. A. Battandier s.n.* (Lectotype, designated here: MPU [MPU006498]!; isolectotype MPU [MPU006497]!). [image of lectotype available at https://plants.jstor.org/stable/viewer/10.5555/al.ap.specimen.mpu006498]

#### Type.

[Algeria]. Djebel Aïssa, rocailles gréseuses près de l’Aïn-Aïssa, 1600 m, [without date], *J. A. Battandier s. n.* (Lectotype, designated here: P [P00083087]!; isolectotype MPU [MPU007787, MPU007788]!). [image of lectotype available at https://plants.jstor.org/stable/viewer/10.5555/al.ap.specimen.p00083087]

#### Notes.

*Verbascumatlanticum* was described by Battandier and Trabut (1889) based on material collected south of Oran, Algeria. In the protologue, the authors did not designate a holotype, nor did they provide data about the herbaria housing the original material. According to Stafleu and Cowan (1976–1988), the plants collected by Battandier and Trabut are kept at Herb. MPU, but a number of duplicates can be found at Herbs P and RAR. We traced three specimens stored at Herb. MPU (MPU007787, MPU007788) and Herb. P (P00083087). The morphology of all three specimens agreed with the original description, and their locality was also in agreement with the locality data given in the protologue. It should also be noted that the specimens housed at Herb. MPU were mounted as two preparations for the same collection – sheets MPU007788 and MPU007787. Cross-labeling indicated that they were a single specimen (see Art. 8.3. of the ICN, Turland et al. 2018). The herbarium sheet P00083087 and those kept at Herb. MPU bore original labels handwritten by Battandier “Université d’Alger / Herbier de l’Afrique du Nord / *Verbascumatlanticum* Batt.! / Type! / Djebel Aïssa, rocailles grèseuses près de l’Aïn-Aïssa, 1600 m, / Leg J. A. Battandier”. Referring to Art. 9.6 of the ICN (Turland et al. 2018), all these specimens should be considered as syntypes. We selected sheet P00083087 as the lectotype, because it was in a better condition than the other Herb. MPU specimens, and part of its features closely agreed with the original description.

In his contributions for the Atlantic Flora, Battandier (1919) described another new species, *V.pseudoblattaria*, thirty years after the description of *V.atlanticum*. The original material of this new species, as referred to by Battandier (1919) in the protologue, was collected at Djebel-M’zi, south of Oran (Algeria). We traced two sheets at Herb. MPU (MPU006498, MPU006497), which are in complete agreement with the protologue and can be considered as original material. The sheet MPU006498 is selected here as the lectotype of the name *V.pseudoblattaria*, since it is in a better condition.

### ﻿ Verbascum ballii

Taxon classificationPlantaeLamialesScrophulariaceae

(Batt.) Hub. -Mor., in Bauhinia 5(1): 10. 1973.

748D0104-66CD-5FEC-B745-5FFA78BDA307

≡ Celsiaballii Batt., in Battandier & Trabut, Fl. Algèrie, Dicot.: 628. 1889 [basionym]. Type: [Algeria]. Oued Biskra, April 1895, *J. A. Battandier, s.n*. (Neotype, designated here: MPU [MPU007789]!, isoneotype MPU [MP007790]!). [image of neotype available at https://plants.jstor.org/stable/viewer/10.5555/al.ap.specimen.mpu007789]. ≡ Celsialaciniatavar.ballii (Batt.) Batt., in Battand. & Trab. Fl. Anal. & Synopt. Alg.-Tun.: 243. 1902. ≡ Verbascumballii (Batt.) Qaiser, in Fl. Libya 88: 26. 1982, comb. nov. superfl.
= CelsiacreticaL.var.cavanillesii auct. Spicil. Fl. Maroc: 584. 1878, Non Celsiacreticavar.cavanillesii Kunze ex Willk., in Willk. & Lange, Prodr. Fl. Hispan. 2: 545. 1870; nec C.cavanillesii Kunze in Flora 29: 698. 1846.
= CelsialaciniataPoir.var.brvipes Barratte, in Bonnet & Barratte Cat. Rais. Pl. Vasc. Tunisie: 311. 1896. Type: not designated.

#### Notes.

When he described this species, Battandier (in Battandier and Trabut 1889) did not specify in which herbarium the type had been deposited. After an in-depth search, we found three sheets at Herb. MPU bearing labels handwritten by Battandier and rewritten by Dr. R. Maire, on which he noted “Type”. The first sheet (MPU007789) bore a label reading “Oued Biskra, Avril 1895, [Battandier’s signature]”, the second one (MP007790) bore a label reading “*Celsiaballii* Batt. / El Kantara / Avril 1895 / *leg. J. A. Battandier* / [Battandier’s signature]/” and the last one (MPU009629) bore a label reading “*Celsiaballii* Batt / C. Aïn-Oumach, montagne / *J. A. Battandier*”. However, these sheets presented two inaccuracies when compared with the protologue: (1) specimens MPU007789 and MP007790 were collected after the protologue was published, and (2) the collection locality indicated on the label of sheet MPU009629 did not match with the one cited in the protologue. Therefore, the “type” notation by Maire on *Celsiaballii* sheets was mistaken. Since no specimen from the original gathering in any institution was traced, a neotype should be designated according to Arts 9.8 of the ICN (Turland et al. 2018). We selected sheet MPU007789 stored at Herb. MPU as a neotype since it was collected and identified by Battandier and display all the morphological features described in the protologue.

### ﻿ Verbascum battandieri

Taxon classificationPlantaeLamialesScrophulariaceae

(Murb.) Hub.-Mor., in Bauhinia 5(1): 10. 1973.

4A14C3CC-C99B-5901-BDE5-58FEC2A66F7A

≡ Celsiabattandieri Murb., in Lunds Univ. Arsskrift, 2 n.f., 22(1): 209.1925. Type: [Algeria]. Algérie occid. Oran, à Santa Cruz, rochers calcaires au-dessus du col, 15 May 1924, *A. Faure, s.n.* (Lectotype, designated here: JE [JE00013725]!; isolectotype: JE [JE00013700, JE00013701, JE00013702, JE00013703]!).[image available at https://plants.jstor.org/stable/viewer/10.5555/al.ap.specimen.je00013725)].
= Celsialaciniata auct., in Battandier & Trabut, Fl. Alg. 1(4): 629. 1890, non C.laciniata Poir., Lam., in Encycl., Suppl. 2: 147. 1811, nec C.laciniata Coss. ex Ball, in J. Linn. Soc., Bot. 16: 585. 1878.
= Celsiabarnadesiivar.mauritanica Pau, in Mem. Mus. Ci. Nat. Barcelona, Ser. Bot. 1(1): 59. 1922 [basionym]. Type: [Morocco]. Riff oriental aux alentours de Zeluan, 2 May 1910, *C. Pau s.n.* (Lectotype designated by Nualart et al. 2018, p. 614): MA [MA108921]!; isolectotypes: BCN [BCN52463]!, G [G00414977]!, LD [LD1967555]!, MA [MA108919]!, MA [MA108920]!, P [P03425530]!). ≡ Celsiamauritanica (Pau) Sennen & Mauricio, in Monde Pl. 3(66–181): 1. 1929.
= Celsiarhiphaea Murb., in Bot. Not. 1945: 109. 1945 [basionym]. Type: [Morocco]. In declivibus calc, littoris rhiphaei, ad pedem Yebel Malmusi (Bocoya), 100 m alt., 4 June 1927, *Font-Quer 565.* B: [n. v.] ([Type specimen is not traced; likely the sheet has been destroyed during the World War II (Hiepko, 1987)]). ≡ Verbascumrhiphaeum (Murb.) Hub.-Mor., in Bauhinia 5(1): 14. 1973.

#### Notes.

In the protologue of this species, Murbeck (1925) cited more than 30 gatherings, and he indicated the herbaria (B, WU, JE) that housed the specimens he examined. In the absence of any indication of a single specimen as the type, all the specimens cited in the protologue can be considered as syntypes according to Art. 9.6 of the ICN (Turland et al. 2018). Following the indications of Murbeck (1925), we only traced five sheets (JE00013700, JE00013701, JE00013702, JE00013703, JE00013725) at Herb. JE. These specimens completely agree with the protologue and can be safely considered as original material. However, as the other sheets stored at Herb. B mentioned by Murbeck were not found, we can assume that they were all destroyed by the fire following bombing by the Allied forces during World War II (see Hiepko, 1987). A detailed examination of the specimen and photos of JE00013725, presumably examined by Murbeck, matched with all the criteria and the description provided in the protologue, so we select it here as the lectotype of the name *Verbascumbattandieri*.

### ﻿ Verbascum blattaria

Taxon classificationPlantaeLamialesScrophulariaceae

L., Sp. Pl. 1: 178. 1753.

3AA2B6F8-94F1-5634-8CB4-592C6B335FC1

= Blattariaalba Mill., Fig. Pl. Gard. Dict. 1: 45. 1760.
= Verbascumglabrum Mill., Gard. Dict. ed. VIII: n. 8 1768.
= Verbascumcordatum Desf., in Fl. Atl. 1: 186. 1798.
= Verbascumrepandum Willd., Enumeratio Plantarum: 226. 1809.
≡ Thapsusblattaria (L.) Raf., in Fl. Tellur. 4: 89. 1838.
= Verbascumblattariavar.albiflorum G.Don, in Gen. Hist. 4: 497 1838. ≡ Verbascumblattariaf.albiflora (G.Don) House, in Bull. New York State Mus. Nat. Hist. 243–244: 45. 1923.
= Verbascumblattariavar.albiflorum Kuntze, in Revis. Gen. Pl. 2: 468.1891, nom. illeg.
= Blattariavulgaris Fourr., in Ann. Soc. Linn. Lyon, N. S. 17: 125. 1869.
= VerbascumblattariaL.var.brevipedicellatum Halácsy. in Ost. bot. Zeitschr. XLII: 419. 1892. Type: [Greece]. Insula Thasos. Limenas, in pratis arenosis in oliveto. 04 June 1891, *P. Sintenis & J. F. N Bornmüller 655* (Lectotype, designated here: LD [LD1393506 image!]; isolectotypes JE [JE00012328 image!], K [K000806399 image!], B [B100278360 image!, B100278359 image!]. [image of lectotype available at https://plants.jstor.org/stable/viewer/10.5555/al.ap.specimen.ld1393506]. **(2)**
= Verbascumblattariavar.crenatum Rouy in Rouy & Foucaud, Fl. France 11: 10. 1909.
= Verbascumrhinanthifolium Davidov in Trav. Soc. Bulg. Sci. Nat. 8: 101. 1915.
= Verbascumcarduifolium Murb. ex. Hayek, Repert. Spec. Nov. Regni Veg. Beih. 30(2): 131. 1929. ≡ Verbascumblattariavar.carduifolium Murb., in Lunds Univ. Arsskrift, 2n.f., 29 (2): 567. 1933. Type: [Greece]. Malakasi; in vinetis, 17 June 1896, *P. Sintenis, 632.* (leolotype, designated here: B [B100278358 image!]; isolectotypes: WU [WU0126534 image!], LD [LD1364911 image!, LD1395023 image!, LD1392908 image!, LD1394623 image!]). [image of lectotype available at https://plants.jstor.org/stable/viewer/10.5555/al.ap.specimen.b_10_0278358]. **(3)**
= Verbascumblattariavar.gracilipes Murb. in Lunds Univ. Arsskrift, 2n.f., 29 (2): 566. 1933. Type: [Greece]. Volo: Portaria, in vinetis, 02 September 1896, *P. Sintenis 1301* (Lectotype, designated here: B [B100278357 image!]; isolectotypes: JE [JE00012330 image!, JE00012331 image!]; WU [WU0126494 image!]). [Greece]. In valle Tempe: prope Papapuli 28–31 Jully 1913, *B. Tuntas, 1954* (Residual syntype: WU [WU0126493]!; In valle Tempe: prope Papapuli 28–31 July 1913, *B. Tuntas, 1955* (Residual syntype: WU [WU0126492 image!]). [image of lectotype available at https://plants.jstor.org/stable/viewer/10.5555/al.ap.specimen.b_10_0278357]. **(4)**
= Verbascumblattariavar.brachycalyx Murb. in Lunds Univ. Arsskrift, 2n.f., 29 (2): 567. 1933. Type: [Greece]. Insula Zakynthos (Zante): in humidis montis Skopos; reg. infer, 4 May 1926, *J. F. N. Bornmüller 1177* (Lectotype, designated here: B [B100278364 image!]; isolectotypes: B [B100278363 image!]). [Greece]. Insula Kephalonia, in olivetis ad Argostoli, 23 May 1926, *J. F. N. Bornmüller 1174* (Residual syntype: JE [JE00012329 image!], S [S10-26942 image!, S12-12718 image!]). [Greece]. Insula Zakynthos (Zante), in herbidum montis Skopos, 04 May 1926, *J. F. N. Bornmüller 1177* (Residual syntype: S [S10-26943 image!]). [GREECE]. Insula Zakynthos (Zante); in olivetis 23 May 1926, *J. F. N. Bornmüller 1174* (Residual syntype: B [B100278367 image!]). [Greece]. Insula Zakynthos (Zante); in olivetis, 02 May 1926, *J. F. N. Bornmüller 1175* (Residual syntype: B [B100278366 image!]). [image of lectotype available at https://plants.jstor.org/stable/viewer/10.5555/al.ap.specimen.b_10_0278364]. **(5)**

#### Type.

(lectotype was designated by Huber-Morath 1971, pg. 143): LINN [Herb. Linn. No. 242.6.]).

#### Notes.

Original material is kept in the Linnaean Herbarium at the Linnean Society of London; an image of the lectotype is available at http://linnean-online.org/1836/.

**(2)** No specimens were cited in the protologue (Halácsy 1892) of VerbascumblattariaL.var.brevipedicellatum. However, according to Murbeck (1933) the description of this taxon was based on material collected by Sintenis and Bornmüller from 1883 to1892 in Greece. We have located five sheets that have been lodged at the following four Herbs: LD (LD1393506), JE (JE00012328), K (K000806399), and B (B100278360, B100278359), the labels’ data of which correspond to those reported by Murbeck (1933). Hence, these specimens are eligible and can be considered as original material. The sheet LD1393506 preserved at Herb. LD is here selected as the lectotype of the name VerbascumblattariaL.var.brevipedicellatum.

**(3)** The name of *Verbascumcarduifolium* was first published by Murbeck (1925: 168), but without a description. Four years later, Hayek (1929) gave the description of *V.carduifolium* Murb. ex. Hayek and mentioned a locality “The.” [as an abbreviation of Thessalía]. Four years later, the taxonomic status of this species has been assessed by Murbeck (1933: 567) and he regarded it as a variety under *Verbascumblattaria* L. In addition to the morphological description, Murbeck (1933) cited an element collected by P. Sintenis viz. “In monte pindo Malakasi, in pratis supra pagum. determ. Leg. P. Sintenis 17 /6/ 1896, n. 632” and he indicated the herbaria (B, WU, LD) that housed the specimens he surveyed. We located six sheets, all of them from the locality given in the protologue, belonging to the Sintenis collections: B (Barcode B100278358), WU (WU0126534) and LD (LD1364911, LD1395023, LD1392908, LD1394623) at Herb. The sheet B100278358 conserved at Herb. B is here designated as lectotype for the name *V.carduifolium*.

**(4)** In the protologue of Verbascumblattariavar.gracilipes, Murbeck (1933) cited 11 gatherings, but he does not provide the name of the herbarium where the original material has been deposited. Six sheets from three different Herbs (B, JE, WU) were traced only: B (Barcode B100278357), JE (JE00012330, JE00012331) and WU (WU0126492, WU0126493, WU0126494). All of these specimens are consistent with the location and diagnosis given in the protologue and can be considered as original material. Since no specimen-type has been designated, all of these specimens can be considered syntypes under Art. 9.6 of the ICN (Turland et al. 2018). The specimen B100278357 preserved at Herb. P is selected here as the lectotype for the name of this variety.

**(5)** In describing Verbascumblattariavar.brachycalyx, Murbeck (1933) referred to eight collections made in five different regions in Greece, but he did not designate a single type for the name. We traced eight specimens in Herbs. B (B100278364, B100278363, B100278367, B100278366), S (S10-26942, S10-26943, S12-12718) and JE (JE00012329) which can be considered original material. We here designate the specimen B100278364 preserved at Herb. B as the lectotype of the name V.blattariavar.brachycalyx since it is well preserved.

### ﻿ Verbascum boerhavii

Taxon classificationPlantaeLamialesScrophulariaceae

L. Sp. Pl. 1: 177. 1753, Syst. Nat. ed. 12, 2: 169. 1767, Mant. Pl.: 45. 1767.

24FB12CA-F107-5DE7-A2AF-61D0BFAB702E

= Verbascummajale DC., in Fl. Franç. 6: 415. 1815.
= Verbascumbicolor Badarò in Brugnat. Giorn. Fis. Dec. II. vii.: 365. 1824.
= Lychnitisboerhavii (L.) Fourr. in Ann. Soc. Linn. Lyon ser. 2 17: 125. 1869.
= Celsiafloccosa Porta, in Nuovo Giorn. Bot. Ital. 19: 313. 1887, nom. illegit., non Benth.
= Vrebascumportae Willk. in Ill. Fl. Hispan. 2: 124. 1888.
= Verbascumboerhaviivar.knochei Benedí, Orell & J.J.Orell in Butll. Inst. Catalana Hist. Nat. 57: 62. 1989.
= Verbascumboerhaviivar.longebracteatum Willk., Suppl. Prodr. Fl. Hispan.: 170. 1893.
= Verbascummajalevar.bicolor (Badarò) Rouy in Rouy & Foucaud, Fl. France 11: 9. 1909.
= Verbascummajalevar.lanceolatum Rouy in Rouy & Foucaud, Fl. France 11: 9. 1909.
= Verbascumboerhaviivar.portae (Willk.) Knoche, Fl. Balear. 2: 367. 1922.
= Verbascumboerhaviif.bicolor (Badarò) Murb., in Acta Univ. Lund. ser. 2 29(2): 159. 1933.
= Verbascumhookerianumvar.pseudocalycinum Maire & Murb. in Bull. Sco. D’Hist. Nat. Afr. Du Nord 18: 84. 1927. Type: [Morocco]. Grand Atlas, Reraya: rocailles porphyriques au-dessous du Tizi-n-Tagherat, 2600–2800 m, 21 July 1922, *R. Maire s.n*. (lectotype, designated here: RAB [RAB030509!]!). [Morocco]. Grand Atlas, Reraya: rocailles porphyriques au-dessous du Tizi-n-Tagherat, 2500–2800 m, 23 July 1922, *R. Maire s.n*. (Residual syntype: MPU [MPU010269!; MPU010270!]). [Morocco]. Grand Atlas, Reraya: rocailles porphyriques au-dessous du Tizi-n-Tagherat, 3000 m, 23 July 1922, *R. Maire s.n*. (Residual syntype: MPU [MPU010268!]). [image of lectotype available at https://plants.jstor.org/stable/viewer/10.5555/al.ap.specimen.rab030509]**(2)**
= Verbascumboerhaviisubsp.portae (Willk.) Malag., Sin. Fl. Ibér. 92: 1465. 1978.

#### Type.

(lectotype designated by Ferrer-Gallego 2014, pg. 253. 2014): LINN [Herb. Linn. No. 242.2]!). (1)

#### Notes.

Original material is kept in the Linnaean Herbarium at the Linnean Society of London; an image of the lectotype is available at http://linneanonline.org/1832/.

**(2)** Maire and Murbeck (in Murbeck 1927) described V.hookerianumvar.pseudocalycinum on the basis of specimens collected from Reraya region in the Great Atlas, Morocco. However, the protologue does not give the name of the herbarium where the original material was deposited. The search for type material brought us to four specimens housed at Herb. RAB (RAB030509) and at Herb. MPU (MPU010269; MPU010270, MPU010268). The sheet RAB030509 stored at herb RAB is here selected as the lectotype for the name V.hookerianumvar.pseudocalycinum.

### ﻿ Verbascum calycinum

Taxon classificationPlantaeLamialesScrophulariaceae

Ball, in J. Bot. 13: 172. 1875.

EE778D88-2DD6-588D-8F3A-9DC2D054E6BB

#### Type.

[Morocco]. High Atlas: rocks of the Ait Mesan valley 1300–1400 m, 13–16 May 1871, *J. Ball, s.n.* (Lectotype, designated here: K [K000410936]!; isolectotype: K [K000410937]!, P [P03420188]!). [image of lectotype available at https://plants.jstor.org/stable/viewer/10.5555/al.ap.specimen.k000410936]

#### Notes.

In the protologue of *Verbascumcalycinum*, Ball (1875) cited one gathering and he mentioned the collection locality reading “Legimus in regione inferiori Atlantis Majoris, convalle Ait Mesanet prope Sketana”. However, he did not quote any herbarium that houses the original material or accession numbers specified, nor the date of collection. According to Stafleu and Cowan (1976–1988), the main collections of Ball are preserved at Herbs E and K, and also some at Herbs OXF and GL. During searches of Ball’s collections from Morocco, three specimens were traced. One of them is kept at Herb. P (P03420188) and another is stored at Herb. K (K000410937) and is mounted on the same sheet with K000410936. Both specimens K000410937 and P03420188 bear a label reading: “Iter Maroccanum. 1871 / *Verbascumcalycinum*, Ball / *Ex regione* inferiori Atlantis Majoris, prope Sketana, alt. 1300–1400 met / Majo 18–19 / *J. A. ball* /”. However, the specimen K000410936 bears a label reading “Ex rupibus arenaceis Atlantis Majoris in convalle Ait Mesan, alt. 1400–2000 met / Majo 13–16 / *J. A. ball* /”. All of those sheets were collected by Ball in the High Atlas, Morocco. As the collection locality of both specimens K000410937 and P03420188 agree closely with the protologue, they can be safely considered as original material under Art. 9.4. of the ICN (Turland et al. 2018). In accordance with Art. 9.3 of ICN (Turland et al. 2018), the sheet K000410937 preserved at Herb. K is selected here as the lectotype, because it shows the best quality of preservation of the important diagnostic features.

### ﻿ Verbascum creticum

Taxon classificationPlantaeLamialesScrophulariaceae

(L.) Cav., in Elench. Pl. Horti Matr. 39. 1803.

2D520523-507F-590F-B4E1-3BDDA864B98E

≡ Celsiacretica L., in Syst. Veg. ed. 13: 470. 1774. Type: [Italy]. Ponae Ital. [Lectotype, designated here: Herb. Linn. No. 774.3 (LINN)! (image available at: http://linnean-online.org/7174/); isolectotype: Linn. No. 774.4 (LINN)! (image available at http://linnean-online.org/7175/)]. **(1)**
= Verbascumlyratum Lam., Encyclop. 4: 223. 1797, nom. illegit., non V.lyratum Pourret, Mém. Acad. Toulouse, ser. 1, 3: 332. 1788 (=V.chaixii Vill).
= Ditoxialyrata Raf., in Précis Découv. Somiol.: 40. 1814, nom. illegit.
= Celsialyrata (Lam.) G.Don, in Gen. Syst. 4: 499. 1837, nom. illegit.
= Thapsandracretica (L.) Griseb., in Spic. Fl. Rumel. 2: 40. 1844.
= Celsiacavanillesii Kunze in Flora 29: 698. 1846.
= Verbascumcreticum (L.) Kuntze, in Revis. Gen. Pl.: 468. 1891, comb. nov. superfl.
= Celsiabalearica Gand. in Fl. Eur. 16: 112. 1889, nom. inval.
= Celsiacreticasubsp.balearica Gand. ex Gand., in Nov. Consp. Fl. Eur.:345. 1910.
= Lasiakelyratum (Lam.) Raf., Fl. Tellur. 4: 89. 1838. nom. illegit.
= Celsiaverbascifolia R.Hern. ex J.J.Rodr., Fl. Menorca: 94. 1901, nom. nud.
= Celsiacreticaf.pallenscens Maire, in Bull. Soc. Hist. Nat. Afrique N. 22: 308. 1931. Type: [Algeria]. Bône, pentes de l’Edough, grès, 600 m., 30 April 1930, *R. Maire s.n* (lectotype, designated here: MPU [MPU002670]!; isolectotype: MPU [MPU002671]!). [image of lectotype available at https://plants.jstor.org/stable/viewer/10.5555/al.ap.specimen.mpu002670]**(2)**

#### Notes.

**(1)**Linnaeus (1774: 470) described *Celsiacretica* in *Species Plantarum* as new nomen specificum legitimum. The diagnosis consists of “*Cretica, C. fol. Inferioribus lyratis, superiofibus ovati samplexi caulibus*” followed by a synonym, “*Blattaria perennis cretica incana, foliis binis conjugatis*” that was cited from Morison (1680: 488). The protologue also includes a morphological description “*Folia pinnato-lyrata alterna: Superiora simpilicis; cordato-amplexicolia. Calyces serrati. Filamenta 4: duobus superioribus pilofis; infeririobus, laevibus antheris que majoribis. Corolla flavae fundo superiore maculis 2. ferrugineis*”. However, the collection locality is not indicated. Seven years later, in his *Supplementum Plantarum Systematis Vegetabilium*, Linnaeus [son] (1781: 281) gave a detailed morphological description of *Celsiacretica*, along with the statement “*Habitat in India, Creta*” followed by the symbol “♂” indicating the gender of the particular organs or individuals within a composite plate, according to Simpson (2010). Moreover, another polynomial “*Verbascum foliis radicalibus ovatis petiolatis, caulinis oblongis sessilimus serratis, subtus tomentosis*” from Miller (1758: 182, ic. t. 273) were cited in synonymy.

Among the original material of the genus *Celsia*, preserved in Linnaean herbaria, three sheets relevant to *Celsiacretica* were found: the first (Herb. Linn. No. 774.3, image available at: http://linnean-online.org/7174/) is annotated at the bottom “*celsia cretica*” and “774.3” at above the right hand of the sheet by Linnaeus. The second specimen (Herb. Linn. No. 774.4, image available at: http://linnean-online.org/7175/) is annotated “*cretica*” at the base and “774.4” at above the right corner of the sheet. Both specimens are clearly showing the characters mentioned in the Linnaeus [son] (1781) diagnosis. After careful examination of the available collections and consideration of all elements in the protologue, the sheet No. 774.3 is the most complete and well conserved, and it is designated here as the lectotype of the name *Verbascumcreticum*. However, the sheet No. 774.4 is selected here as the isolectotype.

**(2)** In the protologue of Celsiacreticaf.pallenscens, Maire (1931) provided the following locality “Bòne, pentes du Mont Edough, 300–700 m” but they did not specify the name of the herbarium where the type material has been stored. We traced three sheets (MPU002670, MPU002671), preserved at Her. MPU which is in complete accord with the protologue. The specimen MPU002670 is here chosen as the lectotype for the name Celsiacreticaf.pallenscens.

### ﻿ Verbascum demnatensis

Taxon classificationPlantaeLamialesScrophulariaceae

(Maire & Murb.) Rankou, in Phytotaxa 78(1): 68. 2013.

0D547273-F0B0-5C20-B8A0-45A68A80982F

≡ Celsiasinuatavar.demnatensis Maire & Murb., in Murbeck, Contr. Fl. Maroc: 40. 1923. Type: [Morocco]: Rocailles calcaires au N. d’El-Arba près Demnat 3 Avril 1921, *R. Maire, s.n.* (Lectotype, designated here: MPU [MPU004004]!; isolectotype P [P00083083]!). [(image available at https://herbier.umontpellier.fr/zoomify/zoomify.php?fichier=MPU004004]
= Celsialyratavar.demnatensis (Maire & Murb.) Murb., in Lund. Univ. Arssk., n. f. 2, 22(1): 199. 1925.
= Celsiademnatensis (Maire & Murb.) Maire in Bull. Soc. Hist. Nat. Afrique N. 29: 438. 1938.
= Verbascumpseudocreticumsubsp.demnatense (Maire & Murb.) Ibn Tattou, in Index Syn. Fl. Afrique N. 5: 304. 2013.

#### Notes.

This taxon was firstly published by Maire and Murberk (1923) as CelsiasinuataCav.var.demnatensis Maire & Murb., and they referred to a plant collected by R. Maire in 1921 near Demnat, Morocco (Maire, 1938). In the protologue, the locality, collector, and collection date were indicated as follows: “Rocailles calcaires au N. d’El-Arba, près Demnat, Dr R. Maire, 3 avril 1921”. As mentioned above, the main Réne Maire herbarium of plants from North Africa is now in Herb. MPU (Stafleu and Cowan 1976–1988). During our research, only two herbarium sheets kept at Herbs MPU (MPU004004) and P (P00083083) were traced, which were all collected by Maire. The sheet MPU004004 contains a plant fragment, with leaves, mature fruits, and some young flowers at the upper part of the inflorescence, and two original labels handwritten by Maire; the first one reads “Université d’Alger / Herbier de l’Afrique du Nord / *Celsiasinuata* Cavon. var. / M. Rocailles calcaires au N. d’El-Arba, près Demnat / 3-4-1921/ Dr R. Maire” and the second label states “*Celsiasinuata* Cavon. var. *a typo differt statura minore calyce ± glandulosa, radice perenni*”. This sheet goes with a “letter” from Maire to Murbeck saying that he considered this new sample to be a new variety of *Celsiasinuata* Cav., and gave a diagnosis. The sheet P00083083 bears a fragment of root with basal leaves and a complete plant with flowers and an original label handwritten by Maire noting the same locality, collection date, and collector as those of the sheet at Herb. MPU. Anyway, these sheets agree with the protologue and are original material; in consequence the sheet MPU004004 preserved at Herb. MPU is selected here as lectotype.

### ﻿ Verbascum dentifolium

Taxon classificationPlantaeLamialesScrophulariaceae

Delile, in Sem. Hort. Bot. Monsp.: 28 1836, Ann. Sci. Nat., Bot., sér. 2, 7: 287 (1837)

0F4BE0A4-EE1A-53F0-96AE-75B603470BB5

= Verbascumgranatense Boiss., in Voy. Bot. Espagne 2: 441. 1841. Type: [Spain]. Alhambra [*P. E. Boissier*] *s.n.* (lectotype, designated by Burdet et al. 1990, pg. 623: G [G00002293]!).
= Verbascumcossonianum Ball, in J. Linn. Soc., Bot. 16: 583. 1878. Locality citation in the Protologue of Type: [Morocco]. Mar. merid. In provincia Mtouga prope castellum gubernatoris, 29 May 1871, *Ball, J.* s.n. Type: Non vidi.
= V.nevadense sensu Batt., Fl. Algérie, Suppl. Phan.: 69. 1910. non Boiss.

#### Type.

[France]. Hortus monspeliensis, Julio 1826, N:10 [*A. R. Delile*] *s.n*. (Lectotype designated here: MPU [MPU020145, MPU020144]!). [France]. Hortus Monspeliensis, July 1831, [*A. R. Delile*] *s.n*. (residual syntype: MPU [MPU020143]!). [images of lectotype available at (image available at https://plants.jstor.org/stable/viewer/10.5555/al.ap.specimen.mpu020144 and at https://plants.jstor.org/stable/viewer/10.5555/al.ap.specimen.mpu020145]

#### Notes.

*Verbascumdentifolium* was described by Delile (1836: 28; 1837: 287) based on material growing in the Montpellier botanical garden from collections made in Port-Juvenal at Montpellier. Port-Juvenal was a place where lots of wool bales were coming to France from the Middle East, introducing many species from seeds trapped in the wool. In his monograph of the genus *Verbascum*, Murbeck (1933) discussed in some detail the distribution and synonyms of a species to which this name has generally been applied, but he did not designate a lectotype for the name *V.dentifolium*. According to Murbeck (1933) the original material is kept at herbarium of Montpellier (MPU), in France. Research in Herb. MPU enabled us to locate two specimens bearing Delile’s handwritten labels and agreeing closely with the protologue’s description. The first one is mounted on two sheets. These two preparations, bearing the Herb. MPU barcodes MPU020144 and MPU020145, have a label reading “*Verbascum*, h. m. Julio 1826, N:10” that is accompanied by two other labels in which Delile gave a description of the species. According to Art. 8.3 of the ICN (Turland et al. 2018), the sheets (MPU020144 and MPU020145) must be considered as a single specimen. The second specimen (MPU020146) bears a label reading “*Verbascum dentifolium*, h. m. Julio 1831”. Since the type is not specified, the two specimens are to be considered as syntypes according to Art. 9.6 of the ICN (Turland et al. 2018). The sheet corresponding to Herb. MPU barcodes MPU020144 and MPU020145 and with the collection date “Julio 1826” is selected here as the lectotype for the name *Verbascumdentifolium*.

### ﻿ Verbascum erosum

Taxon classificationPlantaeLamialesScrophulariaceae

Cav., Elench. Pl. Hort. Matrit.: 38. 1803.

EDC48B90-92E4-524F-8236-7F8F85EBC897

= Celsiasinuata Cav., in Anal. Cienc. Nat. 3: 68. 1801.
= Celsialaciniata Poir., Lam., in Encycl., Suppl. 2: 147. 1811.
= Celsiabarnadesii(Vahl)G.Don fìl.var.baetica Willk., in Ill. Fl. Hispan. 2(14): 55. 1888.
= Celsiajeriaensis Pérez Lara, in Willk. Ill. FI. Hispan. 2(14): 56. 1888, nom. nud.
= Verbascumlaciniatum (Poir.) Kuntze, in Rev. Gen. 469. 1891.
= Celsiabaetica (Willk.) Murb., in Lunds Univ. Arsskrift, n. s. 17 (9): 3. 1921.
= Celsiabaetica (Willk.) Font Quer, in Butll. Inst. Catalana Hist. Nat . 26: 56. 1926. comb. nov. superfl.
= Celsialyratavar.sinuata (Cav.) Maire, in Jahand. & Maire, Cat. Pl. Maroc: 668. 1934.

#### Type.

[Morocco]: Tánger vicinii, [Without date], *Broussonet, s.n*. (lectotype, designated by Benedí and Montserrat 1985, pg.104): MA [MA108913]).

#### Notes.

This species was originally described as *Celsiasinuata* by Cavanilles (1801: 68) based on material collected by Broussonet in Tangier, Morocco (Benedí and Montserrat 1998). When the genus *Celsia* was subsumed in the genus *Verbascum* the epithet *sinuata* could not be used for this species as it already exists in the combination *Verbascumsinuatum* L. for another species. We concur with the conclusion of Benedí and Montserrat (1985) that the correct name for this plant in question is *V.erosum* Cav.

### ﻿ Verbascum faurei

Taxon classificationPlantaeLamialesScrophulariaceae

(Murb.) Hub.-Mor., in Bauhinia 5: 12. 1973.

BDF02933-7253-5713-BC81-6FF4DA293E6B

≡ Celsiafaurei Murb., in Lunds Univ. Arsskrift, 2 n.f., 17(9): 7. 1921. Type: [Algeria]. Oued-Imbert (dépt d’Oran). Lieux rocailleux, 4 Juin 1911, *A. Faure, 304* [Superseded lectotype, selected by Benedí and Montserrat 1997, pg. 168 (Art. 9.19 of the ICN; Turland et al. 2018); lectotype, designated here: LD [LD1244709]!]. [Algeria]. Algérie. Oued-Imbert: Talus de la voie ferrée vers les Lauriers-Roses, 29 Mai 1921, *A. Faure, s.n.* (residual syntype: LD [LD1223485, LD1223785, LD1223665, LD1220485]!). [image of lectotype available at https://plants.jstor.org/stable/viewer/10.5555/al.ap.specimen.ld1244709]

#### Notes.

Murbeck (1921) described *Celsiafaurei* based on collections done by A. Faure in western Algeria. The author in the protologue includes an explicit reference to the locality (Algeria, Oran: Between Imbert River and the railway embankment), with several years for the collection (1911, 1916, 1918, and 1921) and the herbarium where the original material are kept (LD). Benedí and Montserrat (1997) designated the A. Faure material at Herb. MPU as lectotype and that preserved at Herb. LD as isolectotype. However, the material kept at Herb. MPU cannot be considered as original material since the herbarium that housed the type specimens is indicated in the protologue. The lectotype designation by Benedí and Montserrat (1997) is obviously flawed and cannot be considered here because it is in conflict with the protologue according to Art. 9.19 of the ICN (Turland et al. 2018). Pertaining to the original material specification provided in the protologue, five specimens at Herb. LD were traced (LD1223785, LD1220485, LD1223485, LD1223665, and LD1244709) on which are stamped “Type”. In the absence of indicating a single specimen as the holotype, all of the specimens cited in the protologue are to be treated as syntypes (Art. 9.6 of ICN, Turland et al. 2018). The sheet LD1244709 is chosen here as the lectotype, because it shows the best quality of preservation of the important diagnostic features of the taxon. However, the other remaining specimens (LD1223485, LD1223785, LD1223665, LD1220485) traced are referred to as original material following the Art. 9.4 of ICN (Turland et al. 2018).

### ﻿ Verbascum faurei subsp. acanthifolium

Taxon classificationPlantaeLamialesScrophulariaceae

(Pau) Benedí & J.M.Monts., Lagascalia 20: 169. 1997.

03E9E636-E25A-5138-948B-09EAB972EE14

≡ Celsiaacanthifolia Pau in Font Quer. Iter Maroc. n° 566. 1927. Type: [Morocco]. pr. Badú (Atlante rhiphaeo); Hab. in saxosis, solo schistose, 6 July 1927, *P. Font Quer, 566* (lectotype, selected by Benedí and Montserrat 1997, pg. 169, first step “type”, as “*Font Quer 566-1927*”; second step, designated here: BC [BC43694]!; isolectotypes: LD [LD1222710]!, GDA [GDA39155]!, G [G00015117]!, MPU [MPU009627, MPU009628]!, BCN [BCN18029]!, BM [BM000930561]!). [image available at http://psimg.jstor.org/fsi/img/pdf/i0/10.5555/al.ap.specimen.bc43694_normal.pdf]
≡ Celsiaacanthifolia Pau, in Cavanillesia 1: 47. 1928, nom. nud.
≡ Celsiafaureivar.acanthifolia (Pau) Maire in Jahand. & Maire. Cat. PI. Maroc.3: 669 (1934).

#### Notes.

Font Quer (1927) based his species on a collection of plants from the Rif region that he has gathered during his 1927 campaign in Morocco. Font Quer gave the following diagnosis “Folia Acanthi mollis L., superiora oblonga, sinuato-dentata, floribus longe pedunculatis, pedicellis glanduliferis, racemosis; bractae lanceolatae brevissimae, calycis laciniae lanceolatae, margine integro, capsula globosa, obtusa calyce triplo longior” with the collection locality “hab. in saxosis, pr. Badù (Atlante rhiphaeo), 1500 m. alt., solo schistoso, 6 julii”. This protologue has been validly published by Gonzâlez-Bueno (1988). According to Nualart (2017), Benedí and Montserrat (1997) wrongly cited the collection number of the lectotype as “BC436934” instead of “BC43694”. So, in addition to the specimen kept at Herb. BC (BC43694), seven other specimens are in agreement with the description in the protologue. They are housed at Herbs LD (LD1222710), GDA (GDA39155), G (G00015117), MPU (MPU009627, MPU009628), BCN (BCN18029), and BM (BM000930561). According to Art. 9.6 of the ICN (Turland et al. 2018), all of these specimens and those persevered at Herb. BC must be treated as syntypes. The designation by Benedí and Montserrat (1997) can be considered here as a first-step typification (see Art. 9.17 of the ICN, Turland et al. 2018). As the herbarium specimen BC43694 [mounted on three sheets]) kept at Herb. BC is the one showing the best quality of preservation of morphological features described in the protologue, we select it here as second-step lectotype of the name.

The taxonomic status of *Celsiaacanthifolia* has been assessed by some authors. Maire (in Jahandiez and Maire 1934) regarded it as a variety under *Cesliafaurei* Murb. [*C.faureivar.acanthifolia* (Pau) Maire]. Later, Benedi and Montserrat (1997) raised it to the rank of subspecies and they published it as a new combination, Verbascumfaurei(Murb.)Hub.-Morathsubsp.acanthifolium (Pau) Benedí & J.M.Monts.

### ﻿ Verbascum fontanesii

Taxon classificationPlantaeLamialesScrophulariaceae

Benedí, in Anales Jard. Bot. Madrid 60(2): 459. 2003.

3B02CA9D-D841-58D1-82C6-C5829B384F48

= Celsiabetonicifolia Desf., in Flora Atlantica 2: 58. 1798. Type: [Algeria]. In arvis in cultis Algeriae, [without date], *R. L. Desfontaines, s.n.* (Lectotype, designated by Benedí 2003, pg. 459: P [P-DESF]!; isolectotype G [00439692]!).
= Verbascumbetonicifolium (Desf.) Kunze, Revis. Gen. Pl.: 469. 1891, nom. illegit. non V.betonicifolium Desf. in Ann. Mus. Natl. Hist. Nat. 11: 54. 1808.
= Ditoxiabetonicifolia (Desf.) Raf., Prec. Découv. Somiol.: 40. 1814.

#### Notes.

Benedí (2003) indicated as a lectotype the Desfontaines collection kept at Herb. P [P-DESF] but he omitted to mention another sheet of this collection kept at Herb. G (image available at https://plants.jstor.org/stable/viewer/10.5555/al.ap.specimen.g00439692). According to Lasègue (1845) and Stafleu and Cowan (1976–1988), around 600 Flora Atlantica plants was given by Desfontaines to Lemonnier and was acquired by Delessert with the Lemonnier herbarium at Herb. G. In Herb. G we have traced one sheet, with barcode G00439692, that is stamped (Typus). This sample matches the collection locality in the protologue and it is the one that morphologically agrees best with the original description. This specimen bears two labels. The first has “*Celsiabetonicifolia* Desf., Fl. Atl. [Desfontaines’ handwriting]”; the second is a printed label including, name, locality, reference, and a short historical French comment, and is annotated “R-L Desfontaines, Herbier de Barbarie / *Celsiabetonicifolia* Desf. Loc: in arvis ìnculus Algeriae / Desf. Fl. Atl. II, P; 58, Tab. / Série de 600 n^os^ donnée par Desfontaines à L.-G. Lemnier; acquise en 1803 par B. Delessert: revue en 1928 et 1829 par Desfontaines pour servir à illustrer les types décrits dans Flora Atlantica, incorporé en 1916 dans la collection générale de l’Herbier Delessert.- Voyage Lasègue musée botanique de M. Benjamin Delessert. p 60”.

### ﻿ Verbascum gaetulum

Taxon classificationPlantaeLamialesScrophulariaceae

(Maire) Murb., in Bull. Soc. Hist. Nat. Afrique N. 18: 82. 1927.

410C4CD5-1A48-5475-8719-44EB0E9BD449

≡ Verbascumthapsussubsp.gaetulum Maire, in Bull. Soc. Hist. Nat. Afrique N. 9: 182. 1918. Type: Morocco]: Djebel Araïra, lits des oueds, 1400 m, 29 Mai 1918, *R. Maire, s.n.* (Lectotype, designated here: MPU [MPU000364]!; isolectotype: MPU[MPU000365]!). [image of lectotype available at https://plants.jstor.org/stable/viewer/10.5555/al.ap.specimen.mpu000364]
≡ Verbascumsimplexvar.gaetulum (Maire) Maire, in Cat. Pl. Maroc 3: 665. 1934.

#### Notes.

Maire (1918) published the name Verbascumthapsussubsp.gaetulum based on a collection made by himself at the foothills of Araïra Mountain, Morocco. He also observed that this plant was very common in this locality. In the protologue, the author indicated that this plant has an intermediate morphology between *V.thapsus* L. and *V.simplex* Hoffmanns. and Link, but differed from both by its spatulate stigma and its tetrandrous or sub-tetrandrous flowers. Murbeck (1927) raised V.thapsussubsp.gaetulum to species rank. However, a few years later, Maire (in Jahandiez and Maire 1934) classified this taxon with the rank of variety in *V.simplex*, but without any justification for this new recombination. According to African Plant Database (APD) (2022), the accepted name should be *V.gaetulum* (Maire) Murb. despite Ibn Tattou (2007) also considering V.thapsussubsp.gaetulum an accepted taxon.

During our research, we have found two sheets (MPU000364, MPU000365) corresponding to the original material of this taxon preserved at Herb. MPU, where the main collection of Maire is kept. According to Stafleu and Cowan (1976–1988), if the original material of Dr. R. Maire are preserved at Herb. MPU, there are important sets of duplicates at Herbs Al, CAIM, P, and RAB. Since no holotype was specified, the cited specimens are all syntypes under the Art. 9.6 of the ICN (Turland et al. 2018). Therefore, the specimen MPU000364 is selected here as the lectotype, because it is the best original material, more complete and informative than the specimen MPU000365, even if this latter is accompanied by a handwritten detailed morphological description by Maire.

### ﻿ Verbascum hookerianum

Taxon classificationPlantaeLamialesScrophulariaceae

Ball, in J. Linn. Soc. Bot. 16: 584. 1878.

147AFA4D-148C-50AE-B12D-D9EC318ECE9B

= Verbascumtagadirtense Murb., in Murbeck, Contr. Fl. Maroc 2(19): 39. 1923. ≡ Verbascumhookerianumvar.tagadirtense (Murb.) Murb., in Bull. Soc. Hist. Nat. Afrique N. 18: 83. 1927. Type: [Morocco]. Région du Grand Atlas: Tagadirt N’Bourd, c. 1000 m, 09 May 1921, *Sv. Murbeck s.n*. (lectotype, designated here: LD [LD1216036]!; isolectotypes LD [LD1215976]!). **(2)**
= Verbascumhookerianumvar.ballii Murb., in Bull. Soc. Hist. Nat. Afrique N. 18: 83. 1927, nom. nov. superfl.

#### Type.

[Morocco]. South Morocco Greater Atlas, May 1871, *Hooker s. n*. (lectotype, designated here: K [K000410848]!). [image of the lectotype available at http://specimens.kew.org/herbarium/K000410848] (1) .

#### Notes.

*Verbascumhookerianum*, was established by Ball (1878) based on a sample collected by Hooker in the district of Ourika at the foothills of the High Atlas Mountains in Morocco. Ball (1878) in his protologue did not use the term type or mention the herbarium that houses the type specimens. However, in his monograph of genus *Verbascum*, Murbeck (1933) indicated that the original material was kept at Herb. K. During our extensive research, we have traced only one sheet (K000410848) that is totally in agreement with the protologue. This sheet bears a label reading “*Verbascum hookerianum* Nob., South Morocco Greater Atlas, Coll. Dr. Hooker, May 1871” and includes a part of the inflorescence with basal leaf. Since the Herb. K has a single specimen of the Hooker collection, one may argue that the relevant specimen is the holotype. Nevertheless, Ball did not use the term type or mention the name of the herbarium housing the type. Therefore, we here designate the sheet K000410848 as lectotype of the name.

**(2)** The protologue of *V.tagadirtense* comprises a complete description in Latin, followed by the provenance “Région du Grand Atlas-. Pentes broussailleuses à Tagadirt N’Bourd, c. 900 m”, but no indication about the name of the herbarium where the type is preserved (Murbeck 1923). Ten years later, Murbeck (1933), in his monograph of the genus *Verbascum*, indicated that the original material can be found at Herb. LD. During the course of the pursuit, two sheets were traced at Herb. LD which were in accordance with the protologue. The specimen LD1216036 is here selected as a lectotype of *V.tagadirtense*.

### ﻿ Verbascum letourneuxii

Taxon classificationPlantaeLamialesScrophulariaceae

Asch. ex Asch. & Schweinf., Ill. Fl. Egypt 2: 189 & 114. 1887.

34E72831-41BA-5633-98E7-304180014C15

= Verbascumspinosum Delile, Fl. AEg. Illustr.: 55. 1813; non L. Cent. II. plant.: 10. 1756. & Amoen. Acad. IV: 307. 1759.
= Verbascummarniaricum Letournex ap. Barbey Herboris, au Levant: 148. 1882, nom. nud.
= Verbascumtourneuxii Aschers., ap. Barbey 1. C: 182, nom. nud.. Aschers., ap. Aschers. & Schweinf. Illustr. l’I. d. Egypt. in Mém. Instit. Egypt., II: 114. 1887.

#### Type.

[Egypt]. In apricis calcareo-argillosis prope Oum Rakoumi et Matrouka in Marmorica ad limites Cyrenaicae, April 1879, *A. Letourneux, s.n.* (lectotype, designated here: G [G00015113]!; isolectotype: W [W1889-0043225]!, G [G00015111, G00015112, G00015114]!, S [S10-27120]!, K [K000975868]!, P [P03417358, P03417357, P03417360, P03417361]!). [image of lectotype available at https://plants.jstor.org/stable/viewer/10.5555/al.ap.specimen.g00015113].

#### Notes.

Ascherson (in Ascherson and Schweinfurth 1887) described *Verbascumletourneuxii* on the basis of specimens separately collected by Ehrenberg and A. Letourneux from the Alexandria region in the north of Egypt. Within the protologue Ascherson and Schweinfurth (1887) confess that the specimen brought by Ehrenberg only constitutes a skeleton of a plant in fruit, while specimens collected by Letourneux, from a few kilometers from the collection locality of Ehrenberg, are well-developed specimens. However, the authors did not indicate the name of the herbarium housing the type specimen. According to Stafleu and Cowan (1976–1988), the original material of Ehrenberg, Letourneux, Ascherson and Schweinfurth were kept at Herbs B, C, G, K, L, LE, P, S, and W. Nevertheless, the Berlin (B) herbarium was bombed during World War II on the night of 1–2 March 1943 (Hiepko 1987); hence, a good part of the authors’ collection has been lost (Stafleu and Cowan 1976–1988).

Based on the type specification given in the protologue (locality, collector, and collection date), eleven sheets were traced in different herbaria belonging to the Letourneux collections: W (W1889-0043225), G (G00015111, G00015112, G00015113, G00015114), S (S10-27120), K (K000975868), and P (P03417358, P03417357, P03417360, P03417361). Since the type has not been specified, all of the specimens are to be recognized as syntypes according to Art. 9.6 of the ICN (Turland et al. 2018). The sheet G00015113 preserved at Herb. G is here designated as lectotype for the name *V.letourneuxii*, since it is the specimen that shows the best quality of preservation of the important diagnostic features.

### ﻿ Verbascum longirostre

Taxon classificationPlantaeLamialesScrophulariaceae

(Murb.) Hub.-Mor. in Bauhinia 5(1): 13. 1973.

D5D067A2-E7B3-515F-9FED-568340AF80CD

≡ Celsialongirostris Murb., in Lunds Univ. Arsskrift, 2 n.f, 22(1): 190. 1925. Type: [Morocco]. Oudjan, Sud-Ouest du Maroc. 1875, *A. S. Mardochée, s.n.* (lectotype, designated here: P [P03425558]!, isolectotype: P [P03425557]!, K [K000410860, K000410861]!). [Morocco]. Chtouka, Sud-ouest du Maroc, 1875, *A. S. Mardochée, s.n.* (residual syntype: P [P03425553, P03425563]!). [Morocco]. Ida ouchemlal, Sud-ouest du Maroc, 1875, *A. S. Mardochée, s.n.* (residual syntype: P [P03425555, P03425559]!). [Morocco]. Districts de Tazeroualt et Issghivar jusqu’au Si Ahmed ou Moussa 1876, *A. S. Mardochée, s.n* (residual syntype: P [P03425560]!). [Morocco]. Tamelat, Sud-ouest du Maroc, 1875, *A. S. Mardochée, s.n* (residual syntype: P [P03425565]!). [image of lectotype available at https://science.mnhn.fr/institution/mnhn/collection/p/item/p0342555)] **(1)**
= Celsiamaroccana Coss. (in sched) non Ball.

#### Notes.

Murbeck (1925) described this species based on a collection made by A.S. Mardochée, a Moroccan plant specimen collector on account of French botanist Ernest Cosson. In the protologue, Murbeck (1925) cited several collections from south-west of Morocco as original material that are stored at Cosson’s herbarium. The date and collection localities indicated in the protologue are: «Sud-Ouest du Maroc: Chtouka, Ida ouchemlal, Oudjan, Tamelat, 1875, Mardochée; Districts de Tazeroualt et Issghivar jusqu’au Si Ahmed ou Moussa, 1876, Mardochée; Ida Oubouzia, Takoust et Aït zelten, pays montagneux 1876, Mardochée». According to Stafleu and Cowan (1976–1988), the main collections of Cosson are preserved at Herb. P. During our searches we have traced ten specimens collected by Mardochée housed at Herb. P (P03425553, P03425555, P03425557, P03425558, P03425559, P03425560, P03425563, P03425565) and two at Herb. K (K000410860, K000410861). Detailed examination of all of them revealed that the plants and the information on the labels of the samples match well with the protologue description. Since no holotype is indicated in the protologue the ten specimens traced are all syntypes according to Art. 9.6 of the ICN (Turland et al. 2018). Therefore, we select here as the lectotype the sheet P03425558 because it is more complete and agrees best with the original description. This specimen bears two handwritten labels, one by Cosson: “*Celsia maroccana Coss. sp. nov.! Oudjan, Sud-Ouest du Maroc. Mardochée 1875*” and the other bears a 1924 annotation by Murbeck who correctly identified it as *C.longirostris* Murb.

Moreover, as mentioned by Murbeck (1925), Cosson (in sched.) have described this plant in question as a new species beneath the name “*Cesliamaroccana*” accompanied by a handwritten diagnosis written by him on 24 August 1876. However, rendering to Murbeck (1925), Cosson has misinterpreted the plant in question as a new species because at the time mentioned he did not seem to have known that a related species was published a year earlier by Ball (1875: 172) under the name of *Celsiamaroccana*.

### ﻿ Verbascum longirostre var. antiatlantica

Taxon classificationPlantaeLamialesScrophulariaceae

(Emb.) Khamar comb. nov.

621C2E16-4B28-5D69-AB5D-C64B192BF2E1

urn:lsid:ipni.org:names:77318320-1

≡ Celsialongirostrisvar.antiatlantica Emb., in Bull. Soc. Sci. Nat. Maroc 15: 185. 1936. Type: [Morocco]: Anti-Atlas occidental: Falaises gréseuses du Kest, 1400–1500m, 2 May 1936, *L. Emberger, s.n.* (lectotype, designated here: RAB [RAB030634]!; isolectotype: RAB [RAB030632, RAB030633!, RAB030635]!, MPU [MPU006100, MPU006101]!). [image of lectotype available at https://plants.jstor.org/stable/viewer/10.5555/al.ap.specimen.rab030634]

#### Notes.

Emberger (1936) cited a locality in the protologue of Celsialongirostrisvar.antiatlantica: “A A:in praeruptis siliceis montis Kest supra Imdrighis, 15oo m ubi maio floret”, but he did not indicate in which herbarium the specimen(s) were deposited. We have traced six specimens which agree with the protologue and from which to select the lectotype: Four preserved at herb. RAB (RAB030634, RAB030632, RAB030633, RAB030635) and two from Herb; MPU (MPU006100, MPU006101). The sheet RAB030634 kept at Herb. RAB is chosen here as the lectotype of this variety.

### ﻿ Verbascum longirostre var. atlantica

Taxon classificationPlantaeLamialesScrophulariaceae

(Maire) Khamar comb. nov.

25182989-84C4-5078-8E14-96A65DC7D1E5

urn:lsid:ipni.org:names:77318321-1

≡ Celsialongirostrisvar.atlantica Maire, in Maire, Contrib. Étude Fl. Afrique Nord 31: 29. 1940. Type: [Morocco]. in faucibus amnis Dades Atlantis Majoris, solo lapidoso calcareo. 1500m, 21 June 1939, *R. Maire & M. Weiller, 355* (lectotype, designated here: MPU [MPU008974]!; isolectotype: RAB [RAB030628]!). [Morocco]. Grand Atlas: gorges de Tisgi au-dessus des sources du Todra, 11 Juin 1939, *G. Malençon s.n* (Residual syntype: MPU [MPU059325]!) [image of lectotype available at https://plants.jstor.org/stable/viewer/10.5555/al.ap.specimen.mpu008974]

#### Notes.

Celsialongirostrisvar.atlantica was described Maire (1940) based on collections made by Malençon from Tisgi gorges above the sources of Todra and by Maire and Weiller from Dades gorges between 1500–1600 m alt., both of which are situated in the Great Atlas Mountain. Since no holotype was designated in the protologue, these collections should be treated as syntypes in accordance with Art. 9.6 of the ICN (Turland et al. 2018). Three specimens of both collections were traced at Herbs. RAB and MPU. Maire and Weiller’ collection, no. 355 consists of two sheets (RAB030628 and MPU008974), while Malençon’ collection comprises one sheet housed at Herb. MPU (MPU059325). Of these, the specimen MPU008974 housed at Herb. MPU is here selected as the lectotype for the name Celsialongirostrisvar.atlantica due to its high degree of preservation and as it agrees well with the protologue.

### ﻿ Verbascum longirostre var. hoggarica

Taxon classificationPlantaeLamialesScrophulariaceae

(Maire) Khamar comb. nov.

84C739B6-64F4-589B-A6CC-866AEBB8AE65

urn:lsid:ipni.org:names:77318322-1

≡ Celsialongirostrisvar.hoggarica Maire, in Maire, Contrib. Étude Fl. Afrique Nord 31: 29. 1940. Type [Algeria]. In ditione Ahaggar: Tin-Ouzel, granit et roches volcaniques, 2070 m, 31 Mars 1928, *R. Maire, 880* (lectotype, designated here: MPU [MPU004308]!). [Algeria]. In montibus Tefedest, in alveo lapidoso granitico amnis Araghan, 1100 m, 10 Avril 1928, *R. Maire 881* (residual syntype: MPU [MPU004307]!). [Algeria]. Hoggar: Oued Tamanghasset, 28 Février 1933, *J. Lauriol, 363* (residual syntype: MPU [MPU004306]!). [Algeria]. Hoggar: ravins de l’Adrar Haggerane, 03 Mars 1933, *J. Lauriol, s.n.* (residual syntype: MPU [MPU059321]!). [Algeria]. In ditione Ahaggar: Tezzeït, in rupestribus basalticis, 1700–1800 m, 4 Avril 1928, *R. Maire 879* (residual syntype: MPU [MPU004304, MPU004305]!). In ditione [Algeria]. In ditione Ahaggar: in rupestribus graniticis secus amnem Tihaliouin, 2150 m, 22 Martii 1928, *R. Maire 876* (residual syntype: MPU [MPU059319]). [Algeria]. In ditione Ahaggar: Imerera in rupestribus basalticis, 1950–2000 m, 23 Martii 1928, R. *Maire 874* (residual syntype: MPU [MPU059318]!). [Algeria]. In montibus Atokor-n-Ahaggar: in rupestribus graniticis secus amnem Haman, 2000–2001 m, 14 Martii 1928, *R. Maire 875* (residual syntype: MPU [MPU059322]!). [Algeria]. In montibus Atokor-n-Ahaggar: in alvio amnis Temmes-Lezzemt, solo granitico, 2000 m, 15 Martii 1928, *R. Maire 878* (residual syntype: MPU [MPU059306]!). [Algeria]. In ditione Ahaggar: Issekkarassen in rupestribus basalticis, 2070 m 22 Martii 1928, *R. Maire 877* (residual syntype: MPU [MPU059320]!). [image of lectotype available at https://plants.jstor.org/stable/viewer/10.5555/al.ap.specimen.mpu004308]

#### Notes.

When Celsialongirostrisvar.hoggarica was published (Maire 1940), the protologue listed nine collections, but no particular herbarium sheet was chosen as the holotype. We have traced eleven sheets (MPU004304, MPU004305, MPU004306, MPU004307, MPU004308, MPU059306, MPU059318, MPU059319, MPU059320, MPU059321, and MPU05932) preserved at Herb MPU that fit the protologue perfectly. The specimen MPU004308 appears to be the best preserved one and it shows more of the diagnostic features that are described in the protologue Therefore, it is the best candidate for typification and is chosen here as the lectotype for the name C.longirostrisvar.hoggarica according to Arts. 9.3 and 9.4 of the ICN (Turland et al. 2018).

### ﻿ Verbascum lychnitis

Taxon classificationPlantaeLamialesScrophulariaceae

L., Sp. Pl. 1: 177. 1753.

25B4BB12-1FF9-5BAF-A24E-0EB0C9E2BBA2

= Verbascumalbum Mill., in Gardeners Dictionary, Edition 8. London: 3. 1768.
= Blattariaalba Medik., in Vorles. Churpfälz. Phys.-Okon. Ges. 4(1): 230. 1789.
= Verbascumbiebersteinii Besser, Index Seminum (Kalin) 1820: 8. 1820.
= Verbascummicranthum Moretti, Giornale di Fisica II, 5: 111. 1822.
= Verbascumweldenii F.Braun ex Moretti, Giornale di Fisica II, 5: 43. 1822.
= Verbascumincanum Gaudin, in Fl. Helv. 2: 121. 1828.
= Verbascumorchideum Host, in Fl. Austriaca 1: 288. 1827.
= Verbascumleucanthemum Dufour ex Gren. & Godr., in Grenier, Fl. France 2: 552. 1853.
≡ Thapsuslychnitis (L.) Raf., in Flora Telluriana 4: 89. 1838.
≡ Lychnitisalba Fourr., in Annales de la Société Linnéenne de Lyon., 17: 125. 1869.
≡ Lychnitislutea Fourr., in Annales de la Société Linnéenne de Lyon., 17: 125. 1869.
= Verbascumlychnitissubvar.albiflorum Rouy in Rouy & Foucaud, Fl. France 11: 14. 1909.
= Verbascumlychnitissubvar.aureiflorum Rouy in Rouy & Foucaud, Fl. France 11: 14. 1909.
= Verbascumlychnitisvar.foliosum Vayr. in Anales Soc. Esp. Hist. Nat. 30: 553. 1902.
= Verbascumlychnitisvar.longebracteatum Rouy in Rouy & Foucaud, Fl. France 11: 14. 1909.
= Verbascumpyrenaicum Gand., in Dec. Pl. Nov. 2: 9. 1876.
= Verbascumkanitzianum Simonk. & L.Watlz, in Magyar Növényt. Lapok 2: 148. 1878.
= Verbascumlychnitisvar.kanitzianum (Simonk. & L.Watz) Murb., in Lunds Univ. Arsskrift, 2 n.f 29(2): 344. 1933.
= Verbascumlychnitisf.album (Mill.) House in Bull. New York State Mus. Nat. Hist. 243–244: 45. 1923.
= Verbascumlychnitisvar.giganteum Maire, in Mém. Soc. Sci. Nat. Maroc 7: 196. 1924. Type: [Morocco]. Moyen Atlas: Azrou, ravins des cédraies sur basalte et calcaire, 1700 m, 25 Juillet 1921, *R. Maire, s.n*. (Lectotype, designated here: RAB [RAB30546]!; isolectotype: MPU [MPU006911, MPU006912, MPU006913, MPU006910]!, P [P00083084]!). **(2)**
= Verbascumnuriense Sennen, in Diagn. Nouv. 44. 1936. [in shed]

#### Type.

[Habitat in Europae ruderatis cultis.] (Lectotype, designated by Fischer 1997, pg.115: Herb. Clifford: 54, *Verbascum* 2, PM [BM000557980]!).

#### Notes.

Original material is conserved in the Clifford Herbarium at the Natural History Museum of London and an image of the lectotype is available at http://data.nhm.ac.uk/dataset/clifford-herbarium.

**(2)**Maire (1924) described Verbascumlychnitisvar.giganteum on the basis of plant material that he had collected himself in the Azrou cedar forest, Central Middle Atlas, Morocco. However, in the protologue, he did not identify any herbarium sheet as holotype, nor did he give the name of the herbarium where the original material was stored. Six specimens were located through our research at Herbrs RAB (RAB30546), MPU (MPU006910, MPU006911, MPU006912, MPU006913), and P (P00083084) which can be considered original material. The sheet RAB30546 preserved at Herb. RAB is selected here as the lectotype for the name V.lychnitisvar.giganteum since it is in a better condition.

### ﻿ Verbascum mairei

Taxon classificationPlantaeLamialesScrophulariaceae

(Murb.) Hub.-Mor., in Bauhinia 5(1): 13. 1973.

FDF0548A-87F1-58FA-987B-56FEBFD0BF1C

≡ Celsiamairei Murb., Lunds Univ. Arsskrift, 2 n. f., 35(1): 59. 1939. Type: [Morocco]. Tafriat Banks by roadside, 3.000 ft, 10 July 1936, *E.K. Balls, B2792* (lectotype, designated here: S [S10-27049]!; isolectotype: RAB [RAB30643]!). [Morocco]. Moyen Atlas, Roches calcaires à Tizi-n-Ouria (supra Ksiba), 1600 m, 21 Juin 1936, *R. Maire s.n*. (residual syntype: MPU [MPU059317]!). [image of lectotype available at https://plants.jstor.org/stable/viewer/10.5555/al.ap.specimen.s10-27049]

#### Notes.

In the protologue of *Celsiamairei* [≡*Verbascummairei* (Murb.) Hub.-Mor.], Murbeck (1939) cited two gatherings: “Imper. Maroccanum: In rupestribus calcareis Atlantis Medii ad Tizi-n-Ouria (supra Ksiba), 1600 m, R. Maire, 21/6/1936” and “Tafriat, 3000 ft., some 50 or 60 km out of Marrakesh on the road to Ouarzazat across the Great Atlas by Tadert and the Tizi n’ Tichka, E.K. Balls, 10 /7/ 1936, n. 2792”. However, he did not give any information about herbaria housing the original samples, nor did he indicate which one is the holotype.

The search for the original material of *Celsiamairei* led us to discover three sheets kept at Herbs MPU, RAB, and S. The sheet kept at Herb. MPU was collected by R. Maire (Barcode MPU059317), but those found at Herb. S (Barcode S10-270499) and at Herb. RAB (RAB30643) were collected by E.K. Balls. All three specimens found are morphologically in agreement with the original description (Murbeck, 1939). Moreover, collection locations indicated on the labels match those indicated in the protologue. Following Art. 9.6 of the ICN (Turland et al. 2018), these specimens must be considered as syntypes. According to its quality of conservation, the sheet S10-27049 preserved at Herb. S is here selected as the lectotype, because it is well-conserved and shows more diagnostic features described in the protologue. The specimen housed at Herb. RAB is recognized here as an isolectotype.

### ﻿ Verbascum maroccanum

Taxon classificationPlantaeLamialesScrophulariaceae

(Ball) Hub.-Mor., in Bauhinia 5: 13. 1973.

4EB129A4-0AEE-55F3-956E-E371D708B07F

≡ Celsiamaroccana Ball, in Journal of Botany 13: 172. 1875. Type: [Morocco]. South Morocco. Greater Atlas, Seksaoua May 1871, *J. D. Hooker, s.n.* (lectotype, designated here: K [K000410855]!). [Morocco]. South Morocco. Greater Atlas, Milhain, May 1871, *J. D. Hooker, s.n.* (residual syntype: K [K000410856]!). [Morocco]. Near Mogadore. Djebel Hadid, April-May1871, *J. D. Hooker, s.n.* (residual syntype: K [K000410853]!). [Morocco]. South Marocco. Greater Atlas 17 May 1871, *G. Maw, s.n.* (residual syntype: K [K000410857]!). [Morocco]. South Marocco. Greater Atlas, Reraia, May 1871, *J. D. Hooker, s.n.* (residual syntype: K [K000410858]!). [image of lectotype available at https://plants.jstor.org/stable/viewer/10.5555/al.ap.specimen.k000410855]

#### Notes.

This species was described by Ball (1875: 172) from material gathered by himself, J.D. Hooker, and G. Maw during their botanical expedition in Morocco in 1871 (Hooker and Ball 1878; Stafleu and Cowan 1976–1988). Murbeck (1925) indicated that the original material is stored at Herbs K and B. During our research, we have traced five specimens kept at Herb. K and bearing K barcodes as follows: K000410853, K000410855, K000410856, K000410857, K000410858. However, no relevant specimens could be traced at Herb. B, so we have some reasons to think that this material was destroyed during the bombing raid at Berlin in 1943 (Hiepko, 1987). In all the specimens found the collecting localities on labels match those mentioned in the protologue and material morphologically agrees with the original description. Since no holotype was indicated, they are all syntypes according to Art. 9.6 of the ICN (Turland et al. 2018). Hence, among all the original material kept at Herb. K, the specimen bearing the K barcode K000410855 is selected here as the lectotype.

### ﻿ Verbascum masguindalii

Taxon classificationPlantaeLamialesScrophulariaceae

(Pau) Benedí & J.M.Monts., in Collect. Bot. (Barcelona) 16: 108. 1985.

7F709408-1E91-5180-A725-45414AF5866C

= Celsiaramosissima Benth. in DC., Prodr. 10: 244. 1846. Type: [Morocco]. In Mauritania in silva Mamorae, *Durand, s.n.* (Lectotype, designated by Benedí and Montserrat 1985, pg.108: MPU [MAF4378]!).
= Verbascumramosissimum Kuntze, Revisio Generum Plantarum 2: 469. 1891. non Verbascumramosissimum Poir. in Lamarck & Poiret, Encycl. suppl. III: 718, 1813. nec V.ramosissimum DC. in Lam. & DC., Fl. Fr., VI: 416, 1815.
≡ Celsiamasguindalii Pau, Monde des Plantes 66: 1. 1929.
= Verbascumhamidoui Rankou, in Phytotaxa 78(1): 68. 2013.

#### Type.

[Morocco]. Rio Martin, June 1929, *Mas Guindal s.n*. (Lectotype, designated by Benedí and Montserrat 1985, pg. 108: MP [MA108916]!). Image of the lectotype is available at: http://colecciones.rjb.csic.es/#cardAdv.php?CatalogNumber=MA-01-00108916).

#### Notes.

The name of this species was first published by Mas Guindal (1928: 102), but without a description. A year later, Pau (1929) gave the description of *Celsiamasguindalii* Pau based on the specimens collected by Mas Guindal in Río Martín near Tetuán. In their ‘Taxonomic and nomenclatural notes on some species of the genus *Verbascum* L. (incl. *Celsia* L.)’, Benedí and Montserrat (1985) have wrongly cited the lectotype number (MA108989) for *Verbascummasguindalii* (Pau) Benedí & J.M.Monts. instead of the number MA108916 as shown by Nualart (2017). Besides, Nualart et al. (2021) have traced two other eligible sheets (BC89918, BC141503) in Herb. BC. Those two specimens were also collected by Mas Guindal in Río Martín, but without a collection date on the labels. According to Gonzâlez-Bueno and Gomis (2007) and Nualart (2017), Mas Guindal during his visit in Tetouan (Morocco) between 1926 and 1931, has collected many times at this site (Río Martín).

### ﻿ Verbascum maurum

Taxon classificationPlantaeLamialesScrophulariaceae

Maire & Murb., in Lunds Univ. Arsskrift, 2 n.f., 19(1): 35. 1923.

8F59837E-D125-5BF5-A612-690FECD74974

#### Type.

[Morocco]. Region du Grand Atlas. Ourika: rocailles schisteuses le long du torrent, au-dessous de Tagentourt; 1400 m, 14 Juillet 1921, *R. Maire, s.n*. (Lectotype, designated here: MPU [MPU008183]!; isolectotype: MPU [MPU008015]!). [image of lectotype available at https://plants.jstor.org/stable/viewer/10.5555/al.ap.specimen.mpu008183].

#### Notes.

Murbeck (1933), in his monograph of *Verbascum* L., indicated that original material of *Verbascummaurum* was kept at Maire’s herbarium at Herb. MPU. We have only found two sheets of this taxon in Herb. MPU (MPU008015, MPU008183). Both have a handwritten label by Maire bearing the information about the gathering, collector, date, and locality that match the protologue. The specimen MPU008183 is designated here as the lectotype, because it shows more of the diagnostic features described in the protologue.

### ﻿ Verbascum pinnatisectum

Taxon classificationPlantaeLamialesScrophulariaceae

(Batt.) Benedí, in Anales Jard. Bot. Madrid 60(2): 460. 2003.

785ABD20-A7D3-5AB2-B1F8-B56A8C05C9A5

≡ Celsiacreticavar.pinnatisecta Batt. in Bull. Soc. Brot. France 40: 263. 1893. Type: [Algeria]. A. Sersou: Aïn-Sfa, à 40 km de Teniet-el-Had sur la route de Tiaret. Juin 1893, *J.A. Battandier, s.n.* (Lectotype, selected by Benedí 2003, pg. 460, first step “type”; second step, designated here: MPU [MPU006805]!; isolectotype: MPU [MPU005282]! P[P03425758]!). [image of lectotype available at https://plants.jstor.org/stable/viewer/10.5555/al.ap.specimen.mpu006805]
≡ Celsiapinnatisecta (Batt.) Batt. in Batt. & Trab. Fl. Alger. Tunis.: 243. 1904.

#### Notes.

Pursuant to the Art. 9.17 (Turland et al. 2018), the lectotypification of Benedí (2003) could be considered a first-step lectotypification, since he has cited wrongly the number of the lectotype and did not specify exactly which of the two sheets MPU005282 and MPU006805 kept at Herb. MPU as lectotype. In addition to these two sheets stored at Herb. MPU, we have traced another of this gathering kept at Herb. P (barcode P03425758). Hence, the sheet preserved at Herb. MPU that bears barcode “MPU006805” is here designated as the (second-step) lectotype.

### ﻿ Verbascum pseudocreticum

Taxon classificationPlantaeLamialesScrophulariaceae

Benedí & J.M. Monts., in Collect. Bot. (Barcelona) 16: 106. 1985.

63AD3121-B332-528C-BB3F-3351FCD5CA6A

= Celsiasinuata Colla, in Hortus Ripul. App. 2: 344. 1825., non Cav. 1801.
= Celsiacavanillesii Kunze in Flora 29: 698. 1846, nom. illegit., Type: [Spain]: In isthmo Gaditano, III-1846, *M. Willkomm, s.n*. (lectotype, designated by Benedí and Montserrat 1985, pg. 107: COI [COI00042221]!). ≡ Celsiacreticavar.cavanillesii Kunze ex Willk. in Willk. & Lange, Prodr. Fl. Hispan. 2: 545. 1870. Illegitimate name.
= Celsiasinuata sensu Willk., in III. Fl. Hisp. 2(14): 58. 1888, non Celsiasinuata Cav., in Anal. Cieñe. Nat. Madrid 111: 68. 1801.
= Celsialyrata sensu Murb., in Lund. Univ. Arssk., n. f. 2, 22(1): 199. 1925, non Celsialyrata (Lam.) G.Don, in Gen. Hist. 4: 499 1837.

#### Type.

[Spain]. Hab. in regione calida maritima Baeticae: occidentali in arena mobili isthmi Gaditani Inter hortos prope ecclesiam Sancthi Josephi copiose, martii 1845, *M. Willkomm, 536* (lectotype, designated by Benedí and Montserrat 1985, pg. 107: COI [COI00042222]!).

#### Notes.

Original material is conserved at the Herbarium Mediterraneum Pyrenaicum et Canariense of Moritz Willkomm, which is kept at the Herbarium of the Department of Life Sciences of the University of Coimbra (COI). An image of the lectotype is available at http://coicatalogue.uc.pt/specimen/42222.

### ﻿ Verbascum rotundifolium

Taxon classificationPlantaeLamialesScrophulariaceae

Ten., Prod. Fl. Napol. 1: 92. 1815.

BD5339B0-28E4-53EE-819A-74E1426558B4

#### Type.

[Italy]: n.v.

#### Notes.

According to Stafleu and Cowan (1976–1988), the original material of the Italian botanist, Michele Tenore are preserved at Herb. NAP, with further material in Herbs AWH, B-Willd., BASSA, BM, BR, C, CGE, DWG, E, FI, G, H, K, M, MPU, OXF, P, PH, REG, and UPS. However, we could not trace any specimen in any herbarium for this taxon.

### ﻿ Verbascum rotundifolium subsp. rotundifolium

Taxon classificationPlantaeLamialesScrophulariaceae

, in Lunds Univ. Arsskrift, 2n.f. 29 (2): 398. 1933.

6B140D66-3975-5BAB-AA90-D02CAD961D41

= Verbascumnumidicum Pomel, in Nouv. Mat. Fl. Atlantique (1): 95. 1874. Type: [Algeria]. Rochers au sommet du Mécid à Constantine 23 Mai 1857, *S. Choulette 369* (Lectotype designated here: MPU [MPU004892]!, isolectotype JE [JE00013694]!). ≡ Verbascumrotundifoliumvar.numidicum (Pomel) Murb., in Bull. Soc. Hist. Nat. Afrique N. 18: 83. 1927. [image of lectotype available at https://herbier.umontpellier.fr/zoomify/zoomify.php?fichier=MPU004892] **(2)**
= Verbascumkabylianum Debeaux, in Rev. Bot. Bull. Mens. Soc. Franç. de Botan. 8: 265. 1890. Locality citation in the protologue of Type: [Algeria]. In sylyaticis montium kabylie surper. Prope fort-national, ad viam Taourirt-Amokran, 950 m, 6 Junio 1859, *O. Debeaux s.n.*: [n. v.] ≡ Verbascumrotundifoliumvar.kabylianum (Debeaux) Murb. in Bull. Soc. Hist. Nat. Afrique N. 18: 83. 1927.
= Verbascumrotundifoliumvar.castellorum Maire, in Bull. Soc. Hist. Nat. Afrique N. 29:, 438. 1938. Type: [Morocco]. In rupestribus calcareis Atlantis Medii infra Ksiba, 900 m, 22 Juinio 1936, *R. Maire, s.n.* (Lectotype designated here: P [P00083085]!; isolectotype: MPU [MPU004005, MPU004006]!, P [P01167373]!). [image of lectotype available at https://plants.jstor.org/stable/viewer/10.5555/al.ap.specimen.mpu004006]**(3)**

#### Notes.

This taxon was established by Murbeck (1933) under the recombination *V.rotundifolium* subsp. eu-*rotundifolium*. According to Ferguson (1972) and Benedí (2009), this subspecies is distributed in southern Italy, Sicily, Algeria, and Tunisia. However, for Fennane and Ibn Tattou (2005), Ibn Tattou (2007), and Dobignard and Chatelain (2013), this taxon is spontaneous in Morocco. Under its current recombination, V.rotundifoliumsubsp.rotundifolium it is accepted in the Partical flora of Morocco (Fennane and Ibn Tattou 2005, Ibn Tattou, 2007), in the index of synonyms for the flora of North Africa (Dobignard & Chatelain, 2013), and by the APD (African Plant Database 2022).

**(2)**Pomel (1874) described on *Verbascumnumidicum* based on the collections of Choulette exs. [exsiccata] 369, but did not provide collection date nor the name of the herbarium where the original material were housed. After a thorough search, two specimens were found, one at Herb. MPU (MPU004892) and the other at Herb. JE (JE00013694), both of which matched the data provided in the protologue. Thus, the sheet MPU004892 is chosen here as the lectotype of the name *V.numidicum*, because it is better preserved and shows more diagnostic features described in the protologue.

**(3)** The protologue of Verbascumrotundifoliumvar.castellorum (Maire 1938) is composed of a Latin diagnosis, and the details of the collection locality on limestone rocks below Ksiba region at an altitude of about 900 m in the Middle Atlas, Morocco, but there is no mention of the name of the herbarium that houses the original material. Four herbarium sheets deposited in Herbs MPU (MPU004005, MPU004006) and P (P01167373, P00083085) were traced. These specimens can be considered as the original material according to the type details provided in the protologue. The specimen P00083085 persevered at Herb. P is better conserved and best represents the diagnostic features of the taxon given in the protologue; hence it is selected here as the lectotype for the name V.rotundifoliumvar.castellorum.

### ﻿ Verbascum rotundifolium subsp. haenseleri

Taxon classificationPlantaeLamialesScrophulariaceae

(Boiss.) Murb., Bull. Soc. Hist. Nat. Afrique N. 18: 83. 1927.

BA3A6360-F4B9-50A8-8170-6E833F35AB70

≡ Verbascumhaenseleri Boiss., in Voy. Bot. Espagne 2: 442. 1841. Type: [Spain]. Sierra d’Estepona, 1837, [*Boissier*] *s.n.* (lectotype, designated by Burdet et al. 1990, pg. 624: G [G00025472]!). [Spain]. San Anton [*Boissier*] *s.n.* (residual syntype: G [G00025473]!). **(1)**
= Verbascumaurantiacum Coincy, in J. Bot. (Morot) 9: 332. 1895. Type: [Spain]. Espagne, Baza, 6 June 1895, *A. Coincy s.n.* (Lectotype designated here: P [P03808542]!). [image of lectotype available at http://coldb.mnhn.fr/catalognumber/mnhn/p/p03808542] **(2)**
= Verbascumlatesulcatifolium Sennen & Mauricio, in Cat. Fl. Rif Orient.: 84. 1933, nom. nud.
= Verbascumrotundifoliumsubsp.castellanum Murb., in Lunds Univ. Arsskrift, 2n.f. 29 (2): 402. 1933. Type: [Spain]. Ávila; Navalmoral, cerros audossus, 2 June 1863, *E. Bourgeau, s.n.* (Lectotype designated here: MA [MA108794]!), [Spain]. San Agustín de los Reyes, June 1912, *C. Vicioso s.n*. (residual syntype: MA [MA108789]!), [Spain]. Escorial, 15 Juin 1852, *J. Lange, s.n.* (residual syntype: MA [MA108791]!), [Spain]. Prov. Albacete, in pascuis prope Alcaraz, sol. calcareo, 700–800 m, 21 June 1891, *P. Porta* and *G. Rigo, 337* (residual syntype: JE [JE00007506]!). [image of lectotype available at https://plants.jstor.org/stable/viewer/10.5555/al.ap.specimen.ma108794]**(3)**

#### Notes.

**(1)** When, Burdet et al. (1990) selected the lectotype of the name *Verbascumhaenseleri*, they selected the sheet G00025472 as lectotype and sheet G00025473 as a syntype. An image of the lectotype is available at: https://www.ville-ge.ch/musinfo/bd/cjb/chg/result.php?type_search=advanced&lang=en&typecollection=&family=&genus=&species=&infraspecificname=&collector=&nocoll_operateur=%3D&debut_nocoll=&fin_nocoll=&date_operateur=%3D&debut_recolte=&fin_recolte=&country=&locality=&barcode=G00025472.

**(2)**Coincy (1895) in the protologue of *Verbascumaurantiacum* mentioned the collection locality as “Les pentes de la Sierra de Aquila près Baza (prov. de Grenade) le 8 juin 1895, à une hauteur que j’évalue à 1,200 m. environ [The slopes of the Sierra de Aquila near Baza, in the province of Granada, on June 8, 1895, at an altitude that I estimate roughly 1200 m]”. However, no specific herbarium specimen was identified as a holotype, nor was where the original material was housed indicated. According to Stafleu and Cowan (1976–1988), the plants collected by Coincy are preserved at Herb. P, and further material can be found at Herbs B and LY. After conducting exhaustive research in the three different Herbrs (P, B and LY), we located one sheet at Herb. P (P03808542) that is a perfect match with the protologue. Hence, we designate this specimen (P03808542) as the lectotype for the name *Verbascumaurantiacum*.

**(3)** When he described Verbascumrotundifoliumsubsp.castellanum, Murbeck (1933) included in the protologue 14 gatherings that were collected in Spain by diverse botanists, but he did not indicate any herbarium specimen as a holotype. Only four specimens related to the gatherings mentioned in the protologue have been traced. Three of these specimens were found at Herb. MA (MA108791, MA108794, MA108789), and the other specimen was found at Herb. JE (MA108792). (JE00007506). The specimen MA108794 preserved Herb. MA is here designated as lectotype for the name V.rotundifoliumsubsp.castellanum because it is complete and matches the information in the protologue.

### ﻿ Verbascum sinuatum

Taxon classificationPlantaeLamialesScrophulariaceae

L., in Sp. Pl.: 178. 1753.

053BC4E3-BCAA-5477-8FFC-65A50EB2AE62

= Celsiasinuata (L.) Colla, in Hortus Ripul. App. 2: 344. 1826.
≡ Thapsussinuatum (L.) Raf., in Fl. Tellur. 4: 89. 1838.
= Lychnitissinuata (L.) Fourr., in Ann. Soc. Linn. Lyon ser. 2 17: 125. 1869.
= Verbascumsinuatumvar.pallidiflorum Pau, Not. Bot. Fl. Españ. 3: 34. 1889, nom. nud.
= Verbascumsinuatumvar.subulatum Rouy, in Rouy & Foucaud, Fl. France 11: 12. 1909.
= Verbascumtetuanense Pau, in Cavanillesia 1: 143. 1928.
= Verbascumsinuatumf.subsinuatum Pau, in Sched., nom. nud.
= Verbascumarnaizii Sennen, in Diagn. Nouv.: 238. 1936.
= Verbascumsinuatumf.albiflorum M. Silva, in Agron. Lusit. 14: 118. 1952.
= Verbascumsinuatumf.albiflorum Greuter, Matthäs & Risse, in Willdenowia 14: 291. 1984, nom. illegit.

#### Type.

[Protologue locality: Habitat Monspelii, Florentiae] (lectotype, designated by Huber-Morath 1971, pg. 94.: Herb. Linn. No. 242.7 [LINN]!).

#### Notes.

Original material is conserved in the Linnaean Herbarium at the Linnean Society of London and an image of the lectotype is available at http://linnean-online.org/1837/

### ﻿ Verbascum tetrandrum

Taxon classificationPlantaeLamialesScrophulariaceae

Barratte & Murb., in Contrib. Fl. Tunisie 2(14): 62. 1905.

A71493CD-3072-5A0E-BBE3-9980B5EF7374

#### Type.

[Morocco]: Cult. Thurelles, [Cult. Thurelles e. sem. Maroc, 14 July 1877, [*E. Cosson*] *s.n*. (Lectotype, designated here: P [P03429051]!). [Morocco]. Tazeroualt, Sous independant (Maroc), [1874] *Robbin Mardochée* (residual syntype: P [P03429046]!). [image of the lectotype is available at: https://science.mnhn.fr/institution/mnhn/collection/p/item/p03429051?listIndex=288&listCount=7215]

#### Notes.

Barratte and Murbeck’s (in Murbeck 1905) description of *Verbascumtetrandrum* was based on specimens gathered by Rabbi Mardochée near Tazeroualt in the southwest of Morocco. Within the protologue, Barratte and Murbeck (in Murbeck 1905) did not designate any herbarium sheet as holotype, nor the name of the herbarium where the type material was housed. Moreover, the original description was accompanied by a photography of the type with a legend that reads “1-Specimen fructiferum ad Tazeroualt lectum / 2 & 3-Pars caulis foliumque basilare speciminis in Gallia anno 1877 a cl. Cosson culti / 4-Corollae; speciminis culti”. Following this information mentioned in the legend, the type material is formed by the specimens bearing fruits collected by Mardochée and specimens from cultivated grains by Cosson. Murbeck (1933) indicates that the original material was preserved in the Cosson herbarium. During our extensive search, two sheets were found at Herb. P (P03429046, P03429051). All of these specimens agree with the protologue and the accompanied iconography.

The sheet P03429046 contains a fruiting part of an inflorescence and has three labels: an original label handwritten by Cosson “*Verbascum pycnostachyum* nov. sp. / Tazeroualt / Sous independant (Maroc) Robbin Mardochée”; the second is a revision label handwritten by R. Maire in 1921 who re-determined it as “*Verbascusm tetrandrum* Barr & Murb”, and the last label is handwritten by Murbeck in 1926 who put just an exclamation mark (!) indicating that the sheet was verified by him. The sheet P03429051 has an apical part of stem and a basal leaf and two handwritten labels: the first one reads “Verbascum (Celsia) pycnostachyum / Cult. Thurelles / 1877”, and the second is a revision label handwritten by Murbeck in 1924 who re-determined it as “*Verbascusm tetrandrum* Barr & Murb”. All of these specimens should be considered as syntypes as stated by Art. 9.6 of the ICN (Turland et al. 2018). The specimen with barcode P03429051 is here chosen as the lectotype, because it is well preserved and it is the one that shows most of the morphological features in agreement with the original description.

### ﻿ Verbascum tibesticum

Taxon classificationPlantaeLamialesScrophulariaceae

(Quézel) Hub.-Mor., in Bauhinia 5(1): 15. 1973.

D119486B-D6BD-5124-95A3-C54682B8E33E

≡ Celsiatibestica Quézel, in Bull. Soc. Hist. Nat. Afrique N. 48: 95. 1957. Type: [Tchad]. Emi Koussi, Koudou. 2000 m, September-Novembre 1956, *P. Quézel, s n.* (Lectotype, designated here: AIX [AIX000033]!; isolectotype: AIX [AIX000034]!). [image of lectotype available at https://plants.jstor.org/stable/viewer/10.5555/al.ap.specimen.aix000033]

#### Notes.

The type material of this species was collected during Quézel’s expedition to Borkou and to Tibesti in September–November 1956. In the protologue, Quézel (1957) did not designate a holotype nor did he mention the name of the herbarium where the original material was housed. We have traced three specimens at the herbarium of Muséum d’Histoire Naturelle d’Aix-en-Provence (AIX), France. The sheet AIX000033 contains a well conserved plant with mature fruit and rosette and an original label handwritten by P. Quèzel that reads “Mission Botanique de l’Institut de Recherches Sahariennes de l’Université d’Alger au Borkou et au Tibesti / *Celsiatibestica* nov; sp. / Emi Koussi, Koudou. 2000 m / Sept-November 1956, Dr. P. Quézel”. The sheet AIX000034 bears only a rosette and an original label handwritten by P. Quèzel reading “Mission Botanique de l’Institut de Recherches Sahariennes de l’Université d’Alger au Borkou et au Tibesti / *Celsiatibestica* Qz. / Koudou / Sept-November 1956, Dr. P. Quézel”. Both specimens are in accordance with the protologue, and based on Art. 9.6 of the ICN (Turland et al. 2018) both must be treated as syntypes. The sheet bearing the barcode AIX000033 is designated here as the lectotype, because it is in better condition than the other specimen and shows all relevant characters mentioned in the protologue.

### ﻿ Verbascum tripolitanum

Taxon classificationPlantaeLamialesScrophulariaceae

Boiss., in Diagn. Pl. Orient. 12: 9. 1853.

D25635B7-21E2-500A-85A2-CBDFBED0DFB7

#### Type.

[Syria]. Syria mar.: Tripoli, [Jul] 1846, *Ed. Boissier s.n*. (Lectotype, designated here: P [P03287124]!). [image of lectotype available at (Image available at: http://coldb.mnhn.../mnhn/p/p03287124].

#### Notes.

This species was described by Boissier (1853) from material collected at the foot of Trobol mountain in Syria. In the protologue, Boissier (1853) gave information about the collection locality, but he does not provide the name of the herbarium where the original material has been deposited. Boissier (1853) notes that *V.tripolitanum* starts flowering at the beginning of June. According to Stafleu and Cowan (1976–1988) and Jacquemoud (2011), the Boissier herbarium related to the Flora Orientalis account is preserved at Herb. G. However, duplicates of Boissier’s collections could be found in many other herbaria. During our research, we have traced one specimen (barcode P03287124) conserved in Herb. P that is in accordance with the protologue. The sheet P03287124 bears a part of an inflorescence, a basal leaf, and two handwritten labels, the left one reads “*Verbascum tripolitanum* Boiss /Syria Mar: / [Jul] 1846 / Tripoli / (donné par Mr. Boissier / Ed Boiss.)” and the second label on the right corner reads “Herbier Mus. Paris/ herbier tripolinatum / boiss / Syria /”. So this sheet is designated here as the lectotype.

### ﻿ Verbascum zaianense

Taxon classificationPlantaeLamialesScrophulariaceae

(Murb.) Hub.-Mor., in Bauhinia 5(1): 16. 1973.

E203153D-BC50-5094-B8BD-20E1F2E77472

≡ Celsiazaianensis Murb., in Lunds Univ. Arsskrift, 2 n.f., 22(1): 218. 1925. Type: [Morocco]: Entre Aït Lias et Aïn Leuh, 5 Juin 1918, *P. Benoist, 525* (Lectotype, designated here: P [P03425567]!). [image of lectotype available at: http://coldb.mnhn.../mnhn/p/p03425567]

#### Notes.

In the protologue of *Celsiazaianensis*, Murbeck (1923) cited an element collected by Benoist from Zaïan region, between Aït Lais and Ain-leuh, Morocco, and he indicated that type material is preserved at the Cosson Herbarium. The latter collection is housed at Herb. P according to Stafleu and Cowan (1976–1988). The protologue also includes citation of the locality “Entre Aït Lias et Aïn Leuh”, collection date “5 Juin 1918”, and name and number of collector “P. Benoist, 525”. At Herb. P, we have found only one specimen with barcode P03425567 bearing a single plant, and determined by Murbeck as “*Celsia zaianensis* Murb.”. This specimen is morphologically close to the original description. Furthermore, there is an indication on the label indicating a new species (“n. sp.”) and the label has the collection date and collection locality, and collector number matching the information given in the protologue. Since only a single specimen is mentioned by Murbeck (1925), one may argue that the relevant specimen is the holotype. However, Murbeck (1925) did not use the term type. Therefore, we herein designate the sheet P03425567 as lectotype.

## Supplementary Material

XML Treatment for Verbascum atlanticum

XML Treatment for Verbascum ballii

XML Treatment for Verbascum battandieri

XML Treatment for Verbascum blattaria

XML Treatment for Verbascum boerhavii

XML Treatment for Verbascum calycinum

XML Treatment for Verbascum creticum

XML Treatment for Verbascum demnatensis

XML Treatment for Verbascum dentifolium

XML Treatment for Verbascum erosum

XML Treatment for Verbascum faurei

XML Treatment for Verbascum faurei subsp. acanthifolium

XML Treatment for Verbascum fontanesii

XML Treatment for Verbascum gaetulum

XML Treatment for Verbascum hookerianum

XML Treatment for Verbascum letourneuxii

XML Treatment for Verbascum longirostre

XML Treatment for Verbascum longirostre var. antiatlantica

XML Treatment for Verbascum longirostre var. atlantica

XML Treatment for Verbascum longirostre var. hoggarica

XML Treatment for Verbascum lychnitis

XML Treatment for Verbascum mairei

XML Treatment for Verbascum maroccanum

XML Treatment for Verbascum masguindalii

XML Treatment for Verbascum maurum

XML Treatment for Verbascum pinnatisectum

XML Treatment for Verbascum pseudocreticum

XML Treatment for Verbascum rotundifolium

XML Treatment for Verbascum rotundifolium subsp. rotundifolium

XML Treatment for Verbascum rotundifolium subsp. haenseleri

XML Treatment for Verbascum sinuatum

XML Treatment for Verbascum tetrandrum

XML Treatment for Verbascum tibesticum

XML Treatment for Verbascum tripolitanum

XML Treatment for Verbascum zaianense
